# Health and Social Support in the Aftermath of the Maui Wildfires

**DOI:** 10.1001/jamanetworkopen.2025.25430

**Published:** 2025-08-06

**Authors:** Ruben Juarez, Krit Phankitnirundorn, Samia Valeria Ozorio Dutra, Daniela Bond-Smith, Alison G. Lee, Alika K. Maunakea

**Affiliations:** 1 University of Hawaii Economic Research Organization, Honolulu; 2 Department of Economics, University of Hawaii at Manoa, Honolulu; 3 Department of Anatomy, Biochemistry and Physiology, John A. Burns School of Medicine, Honolulu, Hawaii; 4 currently at Faculty of Economics, Thammasat University, Bangkok, Thailand; 5 School of Nursing and Dental Hygiene, University of Hawaii at Manoa, Honolulu, Hawaii; 6 Division of Pulmonary, Critical Care and Sleep Medicine, Department of Medicine, Icahn School of Medicine at Mount Sinai, New York, New York

## Abstract

**Importance:**

Climate-driven wildfires are increasing in frequency and severity, but their association with midterm and long-term health trajectories during the course of recovery remain poorly characterized, particularly in underserved populations.

**Objective:**

To assess the midterm health outcomes of wildfire exposure, including respiratory and mental health, and evaluate the interaction of social support with these health burdens.

**Design, Setting, and Participants:**

The Maui Wildfire Exposure Study (MauiWES) is a community-based, cohort study that enrolled 1174 adults in 2024, 6 to 14 months after the 2023 Maui wildfires. Participants completed exposure assessments, spirometry, biomonitoring, and validated mental health evaluations. Data were accrued from January 26 to September 30, 2024.

**Exposure:**

Living or working in or near areas affected by the August 2023 Maui wildfires.

**Main Outcomes and Measures:**

Primary outcomes included lung function (forced expiratory volume in the first second of expiration [FEV_1_] and forced vital capacity [FVC]), blood pressure, depressive symptoms (10-item Center for Epidemiologic Studies Depression Scale), anxiety (7-item Generalized Anxiety Disorder Scale), and days affected by health issues. Social support was assessed using the Multidimensional Scale of Perceived Social Support.

**Results:**

Among 1174 participants (median age, 47 [IQR, 36-59] years; 684 [59.1%] female; 108 [9.3%] Asian; 239 [20.5%] Filipino; 203 [17.4%] Hispanic or Latino; 238 [20.4%] Native Hawaiian or Other Pacific Islander; 343 [29.4%] White), 150 of 670 (22.4%) exhibited FEV_1_ of less than 80% predicted, 725 of 1152 (62.9%) had high blood pressure (>130/80 mm Hg), and 554 of 1110 (49.9%) reported depressive symptoms. Participants residing within the fire perimeter had significantly lower lung function compared with those outside (FEV_1_ β, −5.97 [95% CI, −10.81 to −1.14] percentage points predicted). Acute wildfire exposure was associated with an increase in days affected by health issues (β, 20.03 [95% CI, 8.87-31.18] days) and elevated depressive symptoms (β, 9.43 [95% CI, 0.61-18.25] points). Higher social support was associated with fewer days affected by health issues (β, −5.08 [95% CI, −8.50 to −1.65] days) but did not mitigate low lung function.

**Conclusions and Relevance:**

In this cohort study of adults affected by the Maui wildfires, substantial midterm cardiopulmonary and psychological health burdens were observed. Higher social support was associated with improved mental health outcomes but did not appear to buffer physiological outcomes such as reduced lung function. These findings highlight the need for disaster response models that integrate clinical surveillance, targeted mental health interventions, and culturally anchored community-based support. As climate-driven disasters intensify, proactive strategies to monitor and mitigate their long-term health effects will be essential to protect vulnerable populations and enhance resilience.

## Key Points

**Question:**

What are the midterm health outcomes among individuals exposed to the 2023 Maui wildfires, and how is social support associated with these outcomes?

**Findings:**

In this cohort study of 1174 adults enrolled 6 to 14 months after the wildfires, 22% demonstrated reduced lung function and 50% screened positive for depressive symptoms. Greater wildfire exposure was associated with increased symptom burden, while higher perceived social support was associated with fewer psychological symptoms.

**Meaning:**

These findings highlight the need for sustained clinical monitoring and community-based mental health support months after the disaster, particularly for individuals with limited social resources.

## Teaser

This cohort study evaluates respiratory, cardiovascular, and mental health outcomes in a diverse population affected by the 2023 Maui wildfires while examining the role of social support.

## Introduction

Wildfires are an increasingly frequent and severe consequence of climate change, affecting health and the environment worldwide. The August 8, 2023, Maui wildfires were the deadliest in the US in more than a century, resulting in more than 100 fatalities, the displacement of more than 8000 residents, and the destruction of all medical infrastructure in Lahaina.^1^ Unlike most wildfires on the US mainland, Hawaii’s geographic isolation presented additional challenges, severely limiting access to emergency health care, essential medications, and personal protective equipment (PPE), including respiratory protection during and after the disaster. The affected population disproportionately consisted of Native Hawaiians, Other Pacific Islanders, and immigrant communities, groups already experiencing significant health disparities, including higher rates of respiratory disease, cardiovascular conditions, and mental health disorders.^2,3,4^

Wildfire exposures pose serious health risks such as increased cardiopulmonary disease, mental health distress, and other chronic diseases with long-term manifestations due to environmental toxins acutely mobilized by fires such as fine particulate matter 2.5 μm or smaller, volatile organic compounds, and heavy metals.^5,6^ However, research has largely focused on short-term smoke exposures, leaving critical gaps in understanding midterm to long-term health effects during the course of recovery. This study addresses these gaps by evaluating respiratory, cardiovascular, and mental health outcomes in a diverse, disaster-affected population while examining the role of social support, a factor often overlooked in disaster resilience.

The rise in climate-driven wildfires underscores the need to understand their health effects,^7,8,9,10^ especially among underserved communities. Thus, we established the Maui Wildfire Exposure Study (MauiWES), a community-based cohort study designed to evaluate midterm to long-term health outcomes of wildfire exposure. MauiWES enrolled 1174 participants between 6 to 14 months post disaster, integrating biomonitoring, spirometry, and validated mental health assessments to examine respiratory, cardiovascular, and psychological outcomes. Additionally, 416 participants (35.4%) completed an extended exposure and health history survey (MauiWES-ExpQ), a novel tool providing further insight into social determinants of health and the role of social support following the disaster.

The urgency of this research is underscored by recent wildfires across the country, which resulted in widespread displacement, severe air quality degradation, and increased cardiopulmonary and mental health risks.^11,12,13^ As climate-driven wildfires become more frequent, findings from the MauiWES reported herein highlight the need for integrated medical and social support strategies to enhance disaster preparedness, protect vulnerable communities, and build long-term resilience.

## Methods

### Cohort Recruitment and Data Collection

The MauiWES was designed as a community-based longitudinal cohort study to assess the midterm to long-term health outcomes of the August 2023 Maui wildfires.^14,15,16,17^ Participants completed a baseline health assessment, and a randomly selected subset (416 [35.4%]) additionally completed the MauiWES-ExpQ between September 19 and October 6, 2024. This structured tool captured wildfire exposure, protective behaviors, symptoms, and social determinants, and is described in greater detail in “Proxies of Exposure” and in the eAppendix in Supplement 1.

Recruitment engaged community partners and sites housing most displaced residents, with multilingual outreach and mobile teams ensuring broad, accessible participation. The final sample was demographically representative of the affected population, although Hispanic participants were slightly overrepresented. This was largely attributable to targeted engagement by trusted community partners, which facilitated participation from historically underrepresented households in Lahaina and the surrounding areas. In-person enrollment included eligibility verification and written informed consent administered in the participants’ preferred languages. All participants received $100 for study completion. The study protocol was approved by the University of Hawaii Institutional Review Board. This study follows the Strengthening the Reporting of Observational Studies in Epidemiology (STROBE) reporting guideline for cohort studies.

Although the MauiWES population reflects the demographic characteristics of the wildfire-affected population, direct comparisons with Maui County are limited due to the lack of accurate postdisaster population data. County-level statistics are used only to contextualize findings. All comparisons should be interpreted cautiously and not as population-level estimates.

### Proxies of Exposure

Wildfire exposure was assessed using a combination of geospatial data and detailed self-report. Fire perimeters were mapped using satellite imagery with validated damage assessments and overlaid with geocoded participant residences (eFigure 1 in Supplement 1). In addition, a randomly selected subsample of 416 participants completed structured questions on smoke, ash, and debris exposure (MauiWES-ExpQ) across 3 timeframes: (1) the acute wildfire period (August 8-11, 2023), (2) the immediate postfire period (August 12 to September 30, 2023), and (3) 5-monthly intervals from October 1, 2023, to September 30, 2024. Four standardized exposure indices were derived: Acute Exposure Score (range, 0-1), Post-Wildfire Exposure Score (range, 0-5), Acute PPE Score (range, 0-1), and Post-Wildfire PPE Score (range, 0-5). These incorporated ordinal frequency scales for exposure intensity and PPE use. Full scoring details are provided in the eMethods and eTable 2 in Supplement 1. All exposure measures relied on self-report, which may introduce recall bias. To reduce this risk, we used phase-specific recall prompts, cross-referenced responses with geospatial fire data, and applied consistent modeling across multiple exposure indices to examine internal coherence and model parsimony (eTables 3-7 in Supplement 1).

### Health Assessments

Pulmonary health was assessed on-site using spirometry (EasyOne; ndd Medical Technologies) in accordance with American Thoracic Society guidelines.^18^ Each participant performed as many as 5 forced expiratory maneuvers, and the best efforts were selected based on reproducibility and quality criteria. Forced expiratory volume in the first second of expiration (FEV_1_), forced vital capacity (FVC), and FEV_1_-FVC ratios were calculated using Global Lung Initiative race-neutral reference equations. Only those graded A, B, or C were retained for analysis to ensure data quality. This process defined the pulmonary MauiWES-ExpQ subgroup (n = 196) used in all lung function analyses. Baseline comparisons indicated that the pulmonary MauiWES-ExpQ subsample was generally similar to the full cohort, with only minor differences observed across select variables (eTable 1 in Supplement 1), reducing concern for systematic bias from missing data. Cardiovascular health was assessed via automated oscillometric blood pressure monitoring, categorized per American Heart Association guidelines.^19^ Body mass index (BMI) was calculated as the weight in kilograms divided by the height in square meters and classified per World Health Organization criteria.^20^ Mental health was assessed using the following validated scales: the 10-item Center for Epidemiologic Studies Depression Scale (CES-D) for depression (scores ≥10 indicating moderate to severe symptoms),^21,22^ the 7-item Generalized Anxiety Disorder Scale (GAD-7) for anxiety (scores ≥10 indicating clinical anxiety),^23^ and the Rosenberg Self-Esteem Scale.^24^ Suicidal ideation was self-reported, triggering immediate clinical follow-up. A licensed psychologist provided on-site support, with referrals as needed.

### Covariate Adjustments

Analyses adjusted for key social and economic determinants, including age, sex, race and ethnicity, BMI, and socioeconomic status as defined by federal poverty thresholds.^25^ Food security was assessed using the US Department of Agriculture 6-item short form, a validated tool for measuring household food insecurity.^26^ Social support was measured using the Multidimensional Scale of Perceived Social Support,^27^ a validated 12-item instrument that captures perceived emotional and instrumental support from family, friends, and significant others. Higher scores indicate greater perceived support. Health-related quality of life was assessed using the Centers for Disease Control and Prevention Healthy Days Measure,^28^ a widely used instrument that captures self-reported physical and mental health during the past 30 days. All covariates were selected based on theoretical relevance and prior literature linking social determinants to health outcomes in disaster settings.

### Race and Ethnicity

Race and ethnicity were self-reported per 1997 Office of Management and Budget standards.^29^ Given Hawaii’s demographic composition, Filipino American individuals were analyzed separately from Asian individuals, while American Indian and Alaska Native and Black or African American individuals were combined due to small sample sizes.

### Statistical Analysis

Descriptive statistics were reported as means with SDs, medians with IQRs, or frequencies with percentages. Group comparisons were performed using χ^2^ tests for categorical variables and 1-way analysis of variance or Kruskal-Wallis tests for continuous variables, as appropriate. Multivariable linear regression models and logistic regression were used to assess associations between wildfire exposure and key health outcomes, including lung function (FEV_1_, FVC, FEV_1_-FVC ratio, and forced expiratory flow, midexpiratory phase), mental health outcomes, reported physical symptoms, cardiovascular health measures, self-reported lung condition, and number of days affected by health issues. All models were also controlled by demographic information (age, race and ethnicity using White as a baseline group, and gender), BMI, preexisting lung conditions, mental health, and other conditions. All missing data were excluded from the analysis. We used interaction effects to model the effect of exposure for different levels of social support instead of stratifying the sample to maintain our sample size and test for a continuous linear association between social support and exposure. The eMethods in Supplement 1 includes additional robustness analyses for all models, using 2 specifications: a fully adjusted model that includes all relevant covariates and a reduced, parsimonious model limited to statistically significant covariates in the fully adjusted model. Analyses that used detailed exposure scores (eg, Acute Exposure Score, PPE Score) were limited to the MauiWES-ExpQ subsample (n = 416), while full-cohort analyses (eg, depressive symptoms, food insecurity) included all 1174 participants. Spirometry analyses were limited to the pulmonary MauiWES-ExpQ subgroup (n = 196), which included only participants with spirometry tests graded A, B, or C. A cohort flow diagram is provided in eFigure 2 in Supplement 1. All statistical analyses were conducted using R, version 4.3.1 (R Program for Statistical Computing). Statistical significance was assessed using 2-sided tests with a significance threshold of *P* < .05.

## Results

### MauiWES Cohort Characteristics and Potential Wildfire Impacts

This cohort study included 1174 participants enrolled 6 to 14 months after the wildfire event. The median age was 47 (IQR, 36-59) years, 684 participants (59.1%) were female, 462 (39.9%) were male, 11 (1.0%) were other, and 17 had data missing or not reported. The participants represented a racially and ethnically diverse population consisting of 108 (9.3%) Asian; 239 (20.5%) Filipino; 203 (17.4%) Hispanic or Latino; 238 (20.4%) Native Hawaiian and Other Pacific Islander, 343 (29.4%) White, and 35 (3.0%) other, with 8 missing or not reporting data. The MauiWES-ExpQ subcohort (416 [35%]) had a similar demographic distribution (Table).

**Table.** Participant Characteristics of the MauiWES Cohort

Characteristic	Full MauiWES cohort (n = 1174)	MauiWES-ExpQ subcohort (n = 416)
Age, median (IQR), y	47 (36-59)	44 (35-59)
Gender, No. (%)		
Male	462 (39.9)	132 (31.7)
Female	684 (59.1)	279 (67.1)
Other	11 (1.0)	5 (1.2)
No. missing or not reported	17	0
Race, No. (%)		
Asian	108 (9.3)	46 (11.1)
Filipino	239 (20.5)	85 (20.5)
Hispanic or Latino	203 (17.4)	51 (12.3)
Native Hawaiian or Other Pacific Islander	238 (20.4)	66 (15.9)
White	343 (29.4)	154 (37.1)
Other^a^	35 (3.0)	13 (3.1)
No. missing or not reported	8	1
Income, No. (%)		
At or above FPL	580 (68.8)	234 (77.2)
Below FPL	263 (31.2)	69 (22.8)
No. missing or not reported	331	113
Food insecurity, No. (%)		
Food secure	495 (47.5)	195 (50.0)
Low food security	303 (29.1)	113 (29.0)
Very low food security	245 (23.5)	82 (21.0)
No. missing or not reported	131	26
Difficulty accessing health care, No. (%)		
Before and since fires	125 (11.3)	33 (8.4)
No difficulty	736 (66.7)	272 (69.2)
Only since fires	243 (22.0)	88 (22.4)
No. missing or not reported	70	23
Participants displaced, No. (%)	645 (55.7)	209 (50.2)
No. missing or not reported	15	0
Employment status, No. (%)		
Employed	693 (59.2)	263 (63.4)
Not employed and looking for work	219 (18.7)	56 (13.5)
Retired or not employed and not looking for work	258 (22.1)	96 (23.1)
No. missing or not reported	4	1
Self-rated health status, No. (%)		
Poor	62 (5.3)	21 (5.1)
Fair	262 (22.6)	82 (20.0)
Good	490 (42.3)	165 (40.1)
Very good	273 (23.6)	120 (29.2)
Excellent	72 (6.2)	23 (5.6)
No. missing or not reported	15	5
Blood pressure, No. (%)		
Normal	315 (27.3)	113 (27.7)
Elevated	112 (9.7)	36 (8.8)
Stage 1 hypertension	490 (42.5)	178 (43.6)
Stage 2 hypertension	235 (20.4)	81 (19.9)
No. missing or not reported	22	8
FEV_1_, median (IQR), % predicted	95 (82-105)	96 (84-106)
No. missing or not reported	504	170
FVC, median (IQR), % predicted	98 (87-108)	97 (87-107)
No. missing or not reported	574	197
10-Item CES-D score, median (IQR)^b^	9 (5-15)	9 (5-15)
Depression, No. (%)		
Normal	556 (50.1)	210 (52.0)
Depressive symptoms	445 (40.1)	149 (36.9)
Highly depressive symptoms	109 (9.8)	45 (11.1)
No. missing or not reported	64	12
Rosenberg Self-Esteem Scale score, median (IQR)^c^	20.0 (16.0-25.0)	21.0 (17.0-26.0)
Self-esteem, No. (%)		
High	244 (22.0)	102 (25.4)
Normal	637 (57.3)	221 (55.1)
Low	230 (20.7)	78 (19.5)
No. missing or not reported	63	15
Suicide ideation, No. (%)	52 (4.6)	17 (4.2)
No. missing or not reported	37	8
GAD-7 score, median (IQR)^d^	6.0 (1.0-10.0)	6.0 (1.0-11.0)
Anxiety, No. (%)		
Minimal	471 (41.1)	172 (41.8)
Mild	362 (31.6)	123 (29.9)
Moderate	174 (15.2)	57 (13.9)
Severe	138 (12.1)	59 (14.4)
No. missing or not reported	29	5
Social support, No. (%)		
High	690 (60.4)	258 (63.4)
Medium	331 (29.0)	102 (25.1)
Low	121 (10.6)	47 (11.5)
No. missing or not reported	32	9
Days affected by health issue, median (IQR)	1 (0-7)	2 (0-7)
No. missing or not reported	2	0

Abbreviations: CES-D Center for Epidemiologic Studies Depression Scale; ExpQ, extended exposure and health history survey; FEV_1_, forced expiratory volume in the first second of expiration; FVC, forced vital capacity; GAD-7, 7-item Generalized Anxiety Disorder Scale; MauiWES, Maui Wildfire Exposure Study.

^a^ Includes American Indian and Alaska Native and Black or African American individuals.

^b^ Scores of 10 or greater indicate moderate to severe symptoms.

^c^ Scores range from 0 to 30, with higher scores indicating greater self-esteem. Scores below 15 are commonly interpreted as indicative of low self-esteem.

^d^ Scores of 10 or greater indicate clinical anxiety.

### Social, Economic, and Mental Health Outcomes

**Figure 1.**
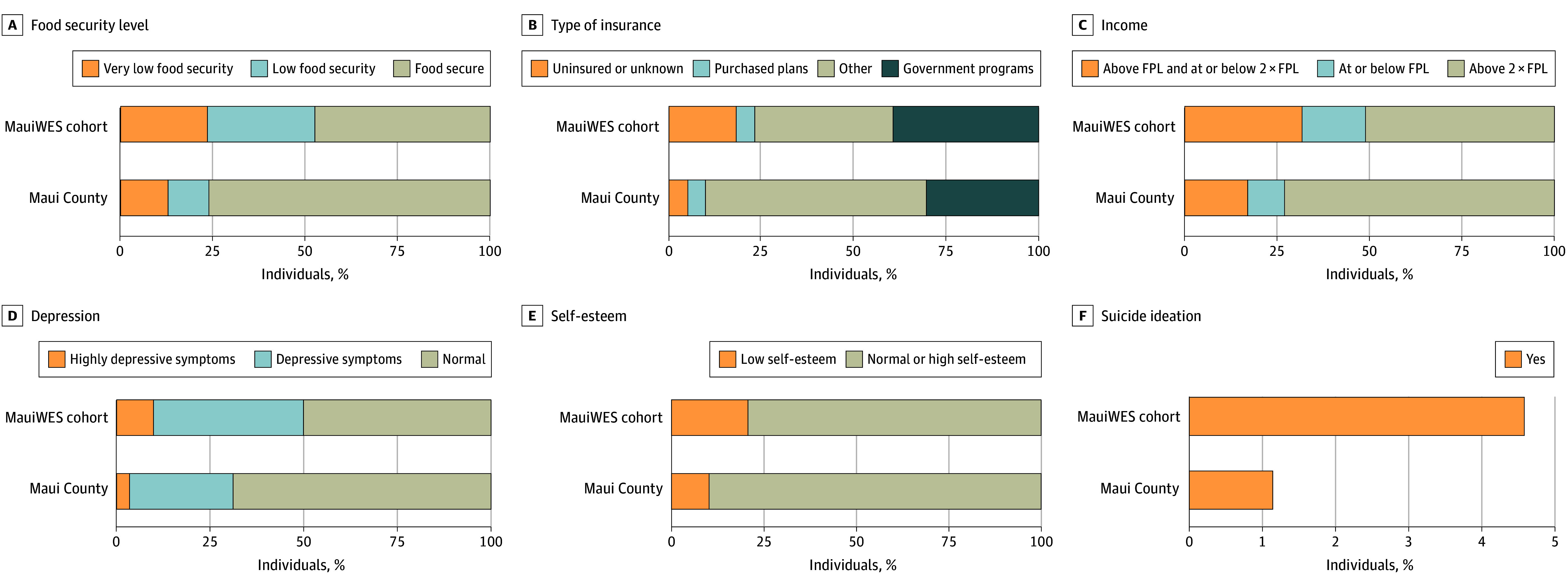
Social and Mental Health Outcomes Selected social and mental health indicators among Maui Wildfire Exposure Study (MauiWES) participants compared with prewildfire Maui County estimates. While the comparison highlights substantial disparities and the psychological toll of the wildfire, direct comparability is limited due to population displacement and the absence of reliable postdisaster denominators. These county-level data are provided for contextual reference only. FPL indicates federal poverty level.

Among the full cohort with available data, 263 of 843 participants (31.2%) lived below the federal poverty level (Figure 1).^30^ Economic hardship was pronounced, with 659 of 843 (78.2%) reporting job or income loss because of the wildfire. Food insecurity was reported by 548 of 1043 participants with data available (52.5%), with 245 (23.5%) experiencing very low food security, more than double the predisaster prevalence for the county of Maui.^31,32^ Participants reported disrupted health care access, with 243 of 1104 (22.0%) reporting new difficulties accessing medical care following the fires compared with 125 (11.3%) before the disaster. Barriers included financial strain (881 of 1174 [75.0%]), lack of transportation (434 of 1174 [37.0%]), and lack of health insurance (176 of 1174 [15.0%]).^30^ Among Hispanic participants, 72 of 203 (35.5%) reported lacking health insurance coverage. Displacement due to the wildfire was associated with reported economic instability, with 477 of 645 (74.0%) of participants reporting household income decline and 309 of 645 (47.9%) unable to return to previous employment. Participants also reported substantial social disruptions, with county estimates of 34% experiencing a loss of close social connections and 21% reporting increased social isolation. Mental health assessments indicated elevated psychological distress among participants. The median CES-D score was 9 (IQR, 5-15), and the median GAD-7 score was 6 (IQR, 1-10). Clinically meaningful depressive symptoms were present in 554 of 1110 participants (49.4%), while 312 of 1145 (27.2%) exhibited clinically relevant anxiety symptoms. Suicidal ideation was reported by 52 of 1137 participants (4.6%), and 230 of 881 (26.1%) reported low self-esteem. Prewildfire mental health prevalence in Maui County indicated baseline depression rates of 30%, low self-esteem prevalence of 13%, and suicidal ideation in less than 1% of the population.^31,32^

### Cardiopulmonary Health Outcomes

**Figure 2.**
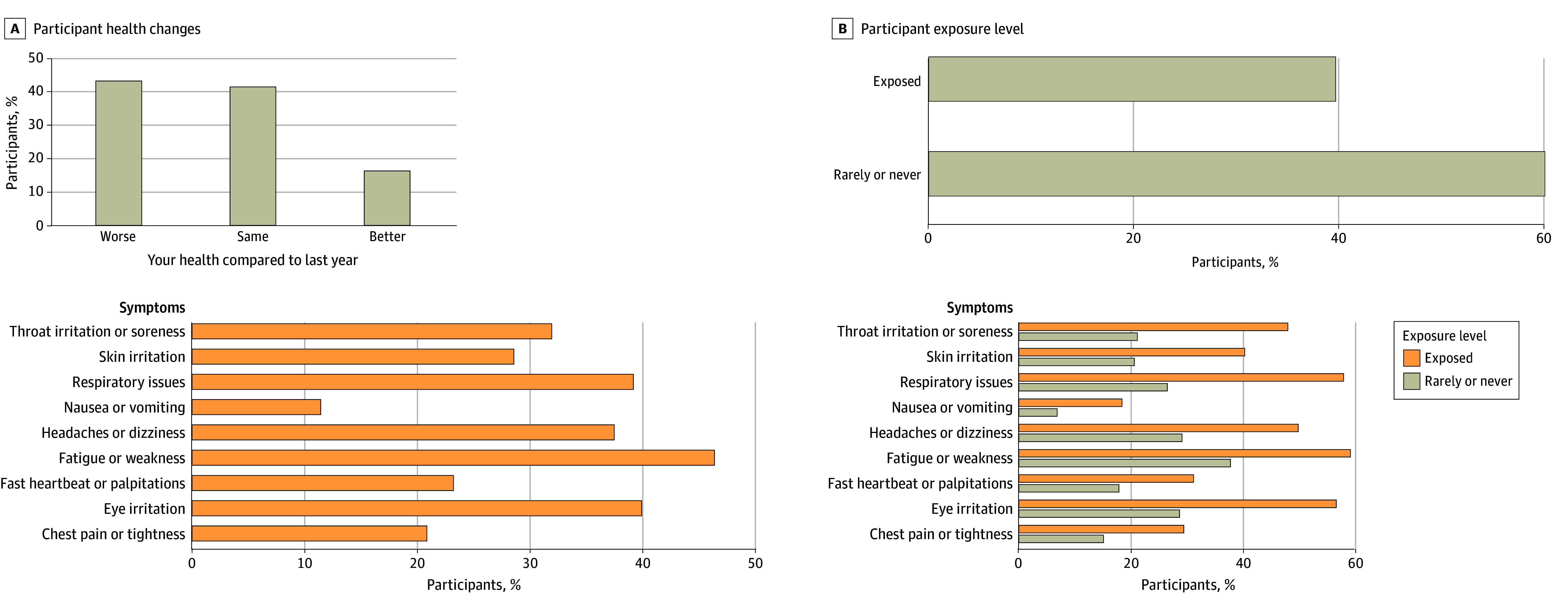
Reported Respiratory Health Indicators by Wildfire Exposure Level The association between wildfire exposure and respiratory symptoms among study participants. Nearly 40% of participants reported a decline in overall health compared with prewildfire conditions, and more than 1 in 4 experienced direct exposure to wildfire ash and airborne contaminants. The figure presents the prevalence of reported symptoms. Stratification highlights self-reported exposure levels associated with symptoms reported.

Among the MauiWES cohort, 495 of 1174 participants (42.2%) reported a decline in self-rated health from the year before the fires, and 459 of 1174 (39.1%) reported significant exposures to fire, ash, and airborne contaminants during and immediately following the disaster. Commonly reported symptoms included fatigue and weakness (554 of 1174 [47.2%]), respiratory symptoms (525 of 1174 [44.7%]), eye irritation (529 of 1174 [45.1%]), and headaches or dizziness (431 of 1174 [36.7%]). Compared with participants reporting lower exposure to wildfire smoke, ash, and debris, those reporting higher exposure exhibited a higher symptom prevalence (Figure 2). Spirometry assessments showed that 150 of 670 participants (22.4%) had below-normal FEV_1_ values, with a median of 95% (IQR, 82%-105%) predicted. Similarly, 72 of 600 (12.0%) exhibited below-normal FVC values, with a median of 98% (IQR, 87%-108%) predicted. Similarly, 72 of 600 (12.0%) exhibited below-normal FVC values, with a median of 98% (IQR, 87%-108%) predicted. Blood pressure assessments indicated that 112 of 1152 individuals (9.7%) had levels classified as elevated (120-129 mm Hg systolic and <80 mm Hg diastolic), 490 of 1152 (42.5%) met criteria for stage 1 hypertension (130-139 mm Hg systolic or 80-89 mm Hg diastolic), and 235 of 1152 (20.4%) met criteria for stage 2 hypertension (≥140 mm Hg systolic or ≥90 mm Hg diastolic).

### Evaluating the Association Between Exposure and Health Outcomes

**Figure 3.**
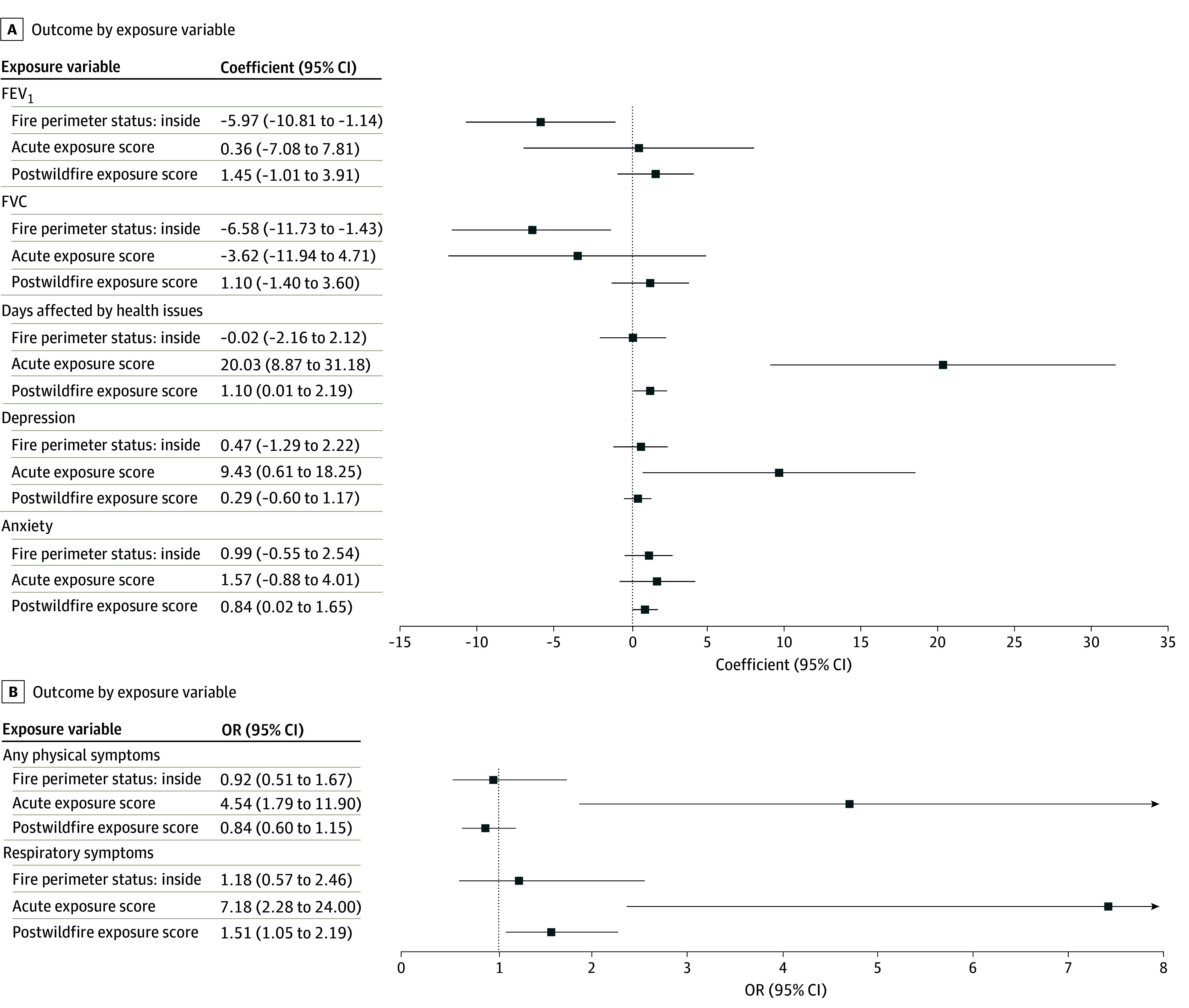
Regression Analysis of Wildfire Exposure and Health Outcomes Multivariable regression analysis examining the association between wildfire exposure and key health outcomes, including lung function (forced expiratory volume in the first second of expiration [FEV_1_] and forced vital capacity [FVC]), days affected by health issues, depression (10-item Center for Epidemiologic Studies Depression Scale), and anxiety (7-item Generalized Anxiety Disorder Scale). Models adjust for demographic, socioeconomic, and preexisting health conditions to account for potential confounders. Findings highlight associations between exposure levels and midterm respiratory and mental health outcomes.

Multivariable regression analyses assessed the association between wildfire exposure and health outcomes using data from MauiWES-ExpQ participants, incorporating granular exposure metrics (Figure 3). Participants with acute wildfire exposure reported significantly more days affected by health issues (β, 20.03 [95% CI, 8.87-31.18] days), whereas those with postwildfire exposure exhibited a more moderate but still significant number of days affected (β, 1.10 [95% CI, 0.01-2.19] days). Residents living within the fire perimeter did not experience a statistically significant difference in the number of days affected compared with those outside the perimeter (β, −0.02 [95% CI, −2.16 to 2.12] days).

Acute wildfire exposure was associated with more depressive symptoms (β, 9.43 [95% CI, 0.61-18.25] points) and anxiety (β, 1.57 [95% CI, −0.88 to 4.01] points), whereas postwildfire exposure was associated with anxiety (β, 0.84 [95% CI, 0.02-1.65] points). Living within the fire perimeter alone was not associated with depression (β, 0.47 [95% CI, −1.29 to 2.22] points) or anxiety (β, 0.99 [95% CI, −0.55 to 2.53] points).

Participants within the fire perimeter exhibited low lung function compared with those outside. FEV_1_ was significantly lower among individuals within the perimeter (β, −5.97 [95% CI, −10.81 to −1.14] percentage points), with similar reductions in FVC (β, −6.58 [95% CI, −11.73 to −1.43] percentage points). Neither acute nor postwildfire exposure showed an association with low lung function. Notably, acute wildfire exposure was associated with significantly higher odds of reporting respiratory symptoms (odds ratio [OR], 7.18 [95% CI, 2.28-24.00]), whereas postwildfire exposure demonstrated a smaller yet significant increase (OR, 1.51 [95% CI, 1.05-2.19]).

### Influence of Preexisting Conditions on Postdisaster Health

Participants with preexisting lung conditions (eg, asthma) had significantly lower lung function post wildfire, with a decrease in FEV_1_ (β, –7.46 [95% CI, –12.32 to –2.59] percentage points) compared with those without lung disease (eFigure 3 in Supplement 1). They were also more likely to report persistent respiratory symptoms (OR, 3.17 [95% CI, 1.54-6.70]), underscoring the respiratory vulnerability of individuals with prior pulmonary conditions.

Preexisting mental health conditions (eg, low self-esteem) were associated with elevated depressive symptoms (β, 2.19 [95% CI, 0.29-4.09] points) and anxiety symptoms (β, 1.80 [95% CI, 0.12-3.49] points). These individuals also reported a greater functional burden, with an increase in days affected by health issues in the past month (β, 2.73 [95% CI, 0.41-5.05]).

### Association Between Social Support and Health Outcomes

**Figure 4.**
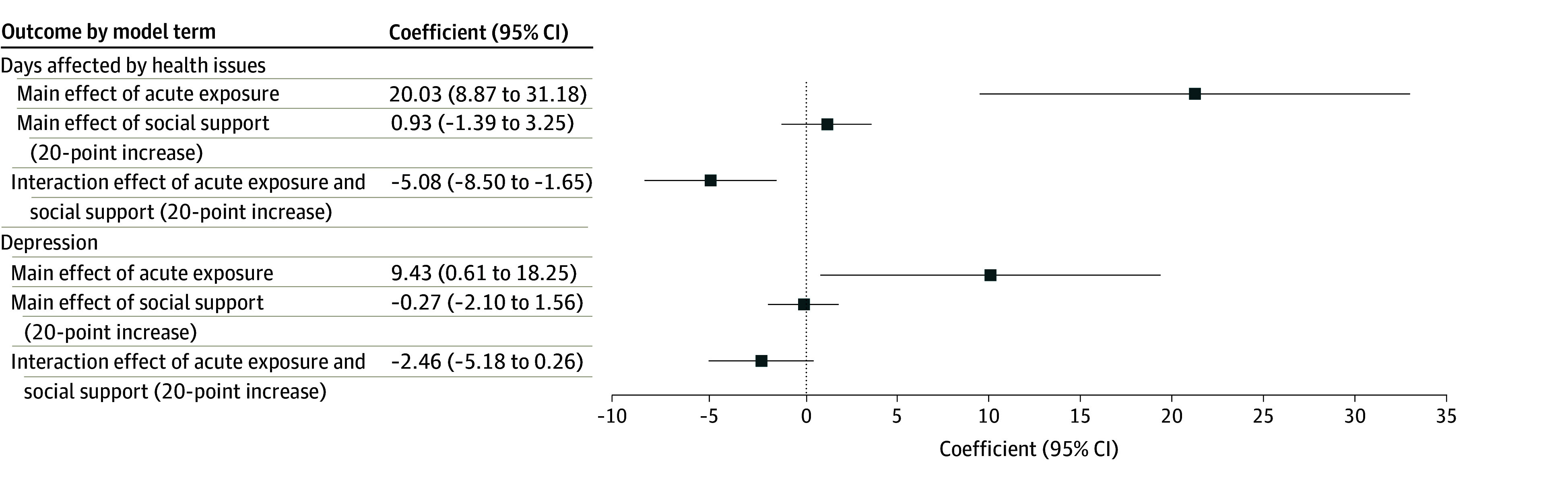
Association Between Social Support and Health Outcomes Multivariable regression analysis assessing the association between social support levels and key health outcomes, including days affected by health issues and depression. Models evaluate both the direct association of social support and its interaction with wildfire exposure to determine its potential protective role.

Regression analyses examined the joint association of social support and wildfire exposure with health outcomes with an interaction effect (Figure 4). Individuals with acute exposure to wildfires experienced a marked increase in days affected by health issues (β, 20.03 [95% CI, 8.87-31.18] days) and in depressive symptoms (β, 9.43 [95% CI, 0.61-18.25] points). In contrast, the main effects of social support—representing its association in the absence of wildfire exposure—were not statistically significant for days affected by health issues (β, 0.93 [95% CI, −1.39 to 3.25] days) or depressive symptoms (β, −0.27 [95% CI, −2.10 to 1.56] points). Notably, the interaction term indicated that higher perceived social support was associated with fewer adverse health symptoms among individuals with greater wildfire exposure. Specifically, exposed individuals who had a 20-point higher social support score, which corresponded approximately to the SD of this variable in the sample, experienced about 5 fewer days affected by health issues (β, −5.08 [95% CI, −8.50 to −1.65] days) and showed fewer depressive symptoms (β, −2.46 [95% CI, −5.18 to 0.26] points). However, social support was not associated with improved lung function among participants with higher wildfire exposure. These findings suggest that while greater perceived social support is linked to better psychological outcomes, it may not extend to physiological outcomes such as lung function. This pattern may reflect the limited influence of psychosocial resources on outcomes that are more directly tied to environmental exposure or barriers to accessing respiratory care.

### Supplementary Analysis

Supplementary analyses explored specific outcomes in detail. eTable 3 in Supplement 1 presents the association between wildfire exposure and self-reported physical and respiratory symptoms. eTable 4 in Supplement 1 examines how wildfire exposure was associated with objective lung function measures, including FEV_1_ and FVC. eTable 5 in Supplement 1 models the number of days participants experienced health-related disruptions as a function of exposure. eTable 6 in Supplement 1 reports regression analyses linking wildfire exposure with depression and anxiety symptom severity. eTable 7 in Supplement 1 assesses the association between wildfire exposure and the prevalence of hypertension. eFigure 4 and eResults in Supplement 1 show that significant racial and ethnic disparities persisted in postwildfire lung function and depression scores, even after adjusting for demographic and socioeconomic factors. These results consistently show that acute exposure during the fires was associated with the most adverse physical and mental health outcomes, while postfire exposure and PPE use showed less or no association.

## Discussion

### Key Findings and Public Health Implications

The midterm to long-term health effects of human exposure to wildfires remain poorly understood. This novel cohort study provides a broad assessment, combining surveys with biological data from a sizable and racially and ethnically diverse cohort of 1174 adults. While we present limited comparisons to Maui County statistics for context, direct comparability is constrained. The wildfires substantially altered the population through displacement and outmigration, precluding accurate postdisaster denominators. These comparisons are not intended to reflect countywide estimates. This study therefore offers, to our knowledge, the first midterm (6-14 months) evaluation of wildfire-related health outcomes in a diverse, historically underserved population in the US.

Nearly half of the MauiWES participants reported persistent respiratory symptoms, and living within the fire perimeter was associated with both higher odds of reporting these symptoms and lower measured lung function. These findings align with prior studies linking wildfire smoke exposure to pulmonary dysfunction and long-term respiratory disease.^33,34,35,36^ Spirometry results further confirmed reduced lung function among exposed individuals, alongside reports of new-onset hypertension and symptoms suggestive of autonomic dysregulation. Together, these outcomes underscore the need for sustained cardiopulmonary surveillance and targeted clinical follow-up in postwildfire settings. Mental health burdens were also substantial, with depressive symptoms exceeding historical prewildfire estimates.^31,32^ The cumulative effects of social disruption, displacement, and economic instability likely intensified psychological distress, reinforcing the critical need for trauma-informed mental health services in disaster recovery frameworks.^37,38,39^

### Risk and Resilience in Disaster-Related Health Outcomes

Participants with preexisting conditions consistently reported poorer physical and mental health as well as more challenges with daily activities following the disaster. These associations are illustrated in eFigure 3 in Supplement 1, which shows consistent adverse outcomes across multiple health indicators among those with preexisting respiratory, mental health, or chronic conditions. Higher social support was linked to better mental health during recovery but was not associated with lung function, underscoring the need for clinical follow-up. Social support was measured using the Multidimensional Scale of Perceived Social Support, which reflects emotional and interpersonal support but not community-based assistance. To strengthen resilience against future disasters, our findings suggest that recovery strategies would benefit by integrating both emotional and tangible support services such as financial aid, housing stability, and culturally grounded care. Similarly, prioritizing individuals with chronic health conditions through cardiopulmonary care and trauma-informed, community-based mental health services remains an essential component.

### Challenges in Assessing Wildfire-Related Health Outcomes

Assessment of wildfire-related health outcomes presents challenges due to variable exposures, delayed data collection, intrinsic interindividual variability, and reliance on self-reported measures. In our study, self-reported exposure via the MauiWES-ExpQ was associated with greater mental health symptoms than with objective outcomes like lung function. This may reflect shared method variance, where individuals experiencing greater distress also perceive or report higher exposure. Alternatively, it could indicate limitations in geographic or objective exposure metrics to capture subjective experience. These findings underscore the need for future work validating multidimensional exposure tools and incorporating environmental monitoring and biomarker data.

### Limitations

This study has several limitations. First, its cross-sectional design limits causal inference, and unmeasured confounding may persist despite covariate adjustment. Second, exposure and health outcome data relied on self-report, introducing potential recall bias, especially among participants experiencing greater hardship. To mitigate this, we used temporally anchored recall in the MauiWES-ExpQ and supplemented self-reports with geospatial fire data. Sensitivity analyses supported the consistency of exposure metrics across domains. Third, spirometry was only performed at intake and was limited to tests with quality grades of A to C (approximately 60% of participants). While this may introduce bias, included participants were demographically representative of the full cohort. Fourth, the absence of a formal unexposed control group limits interpretation of comparisons to historical or census data, which differ in sampling and timing. Fifth, sample size limitations in the MauiWES-ExpQ and spirometry subsamples may have reduced power to detect smaller or more nuanced associations. Several regression models yielded wide 95% CIs, suggesting statistical imprecision. While these sample sizes reflect substantial postdisaster community engagement, future follow-up studies with larger and more diverse populations will be important to validate and extend these findings. Finally, convenience sampling may limit generalizability, and this analysis reflects only midterm outcomes. Long-term follow-up in a diverse and representative population is needed to assess chronic disease development and sustained mental health effects.

## Conclusions

In this cohort study of wildfire-affected adults in Maui, we show the growing health toll of climate-driven disasters and the need for proactive, community-centered responses. Equitable recovery requires culturally tailored care, long-term health surveillance, and investment in local resilience. Our findings call for a shift from reactive disaster relief to integrated strategies that prioritize vulnerable populations and address physical and mental health trajectories following wildfire exposures, along with increasing capacity to address preexisting public health conditions in such populations to strengthen community resilience against future disasters.

## Notes

Supplement 1. **eAppendix.** Maui Wildfire Exposure Study Organization **eMethods.** Eligibility Criteria and Wildfire Exposure Score Computation and Methodology **eFigure 1.** Maui Wildfire Perimeter and Participant Locations **eFigure 2.** Participant Flow Diagram for the MauiWES Dataset **eTable 1.** Participant Characteristics of the MauiWES Cohort **eTable 2.** Exposure Characteristics of the MauiWES-ExpQ **eTable 3.** Association Between Wildfire Exposure and Reported Physical and Respiratory Symptoms **eTable 4.** Association Between Wildfire Exposure and Lung Function Parameters **eTable 5.** Association Between Wildfire Exposure and Days Impacted by Health Issues **eTable 6.** Association Between Wildfire Exposure and Mental Health Outcomes **eTable 7.** Association Between Wildfire Exposure and Hypertension Risk **eFigure 3.** Regression Analysis of Pre-existing Conditions and Health Outcomes **eResults.** Race and Ethnic Disparities in Health Outcomes **eFigure 4.** Racial and Ethnic Disparities in Postwildfire Health Outcomes
eReferences.

Supplement 2.
Data Sharing Statement
