# Supplementary material for: Health and Social Support in the Aftermath of the Maui Wildfires
Source: JAMA Netw Open. 2025 Aug 6;8(8):e2525430. doi: 10.1001/jamanetworkopen.2025.25430 (PMC12329611; doi:10.1001/jamanetworkopen.2025.25430)
Supplement: Supplement 1. — eAppendix. Maui Wildfire Exposure Study Organization eMethods. Eligibility Criteria and Wildfire Exposure Score Computation and Methodology eFigure 1. Maui Wildfire Perimeter and Participant Locations eFigure 2. Participant Flow Diagram for the MauiWES Dataset eTable 1. Participant Characteristics of the MauiWES Cohort eTable 2. Exposure Characteristics of the MauiWES-ExpQ eTable 3. Association Between Wildfire Exposure and Reported Physical and Respiratory Symptoms eTable 4. Association Between Wildfire Exposure and Lung Function Parameters eTable 5. Association Between Wildfire Exposure and Days Impacted by Health Issues eTable 6. Association Between Wildfire Exposure and Mental Health Outcomes eTable 7. Association Between Wildfire Exposure and Hypertension Risk eFigure 3. Regression Analysis of Pre-existing Conditions and Health Outcomes eResults. Race and Ethnic Disparities in Health Outcomes eFigure 4. Racial and Ethnic Disparities in Postwildfire Health Outcomes eReferences. [file jamanetwopen-e2525430-s001.pdf]

Supplementary Online Content

Juarez R, Phankitnirundorn K, Ozorio Dutra SV, Bond-Smith D, Lee AG, Maunakea AK. Health and social support in the aftermath of the Maui wildfires. *JAMA Netw Open.* 2025;8(8):e2525430. doi:10.1001/jamanetworkopen.2025.25430

eAppendix. Maui Wildfire Exposure Study Organization
eMethods. Eligibility Criteria and Wildfire Exposure Score Computation and Methodology
eFigure 1. Maui Wildfire Perimeter and Participant Locations
eFigure 2. Participant Flow Diagram for the MauiWES Dataset
eTable 1. Participant Characteristics of the MauiWES Cohort
eTable 2. Exposure Characteristics of the MauiWES-ExpQ
eTable 3. Association Between Wildfire Exposure and Reported Physical and Respiratory Symptoms
eTable 4. Association Between Wildfire Exposure and Lung Function Parameters
eTable 5. Association Between Wildfire Exposure and Days Impacted by Health Issues
eTable 6. Association Between Wildfire Exposure and Mental Health Outcomes
eTable 7. Association Between Wildfire Exposure and Hypertension Risk
eFigure 3. Regression Analysis of Pre-existing Conditions and Health Outcomes
eResults. Race and Ethnic Disparities in Health Outcomes
eFigure 4. Racial and Ethnic Disparities in Postwildfire Health Outcomes
eReferences,

This supplementary material has been provided by the authors to give readers additional information about their work.

© 2025 Juarez R et al. *JAMA Network Open*.

eAppendix. Maui Wildfire Exposure Study Organization

MauiWES operates under a comprehensive, four-pronged organizational structure that ensures a scientifically rigorous, community-driven, and policy-impactful approach to wildfire disaster research and survivor support. This structure integrates statewide and national expertise, local leadership, and key funding partners, reinforcing our ability to deliver meaningful health and policy interventions for affected communities.

The four-pronged MauiWES organizational structure ensures that wildfire survivors receive comprehensive support through scientific expertise, community leadership, national collaborations, and robust funding mechanisms. This integrated approach strengthens the study's ability to generate evidence-based solutions, influence policy, and enhance long-term disaster resilience efforts.

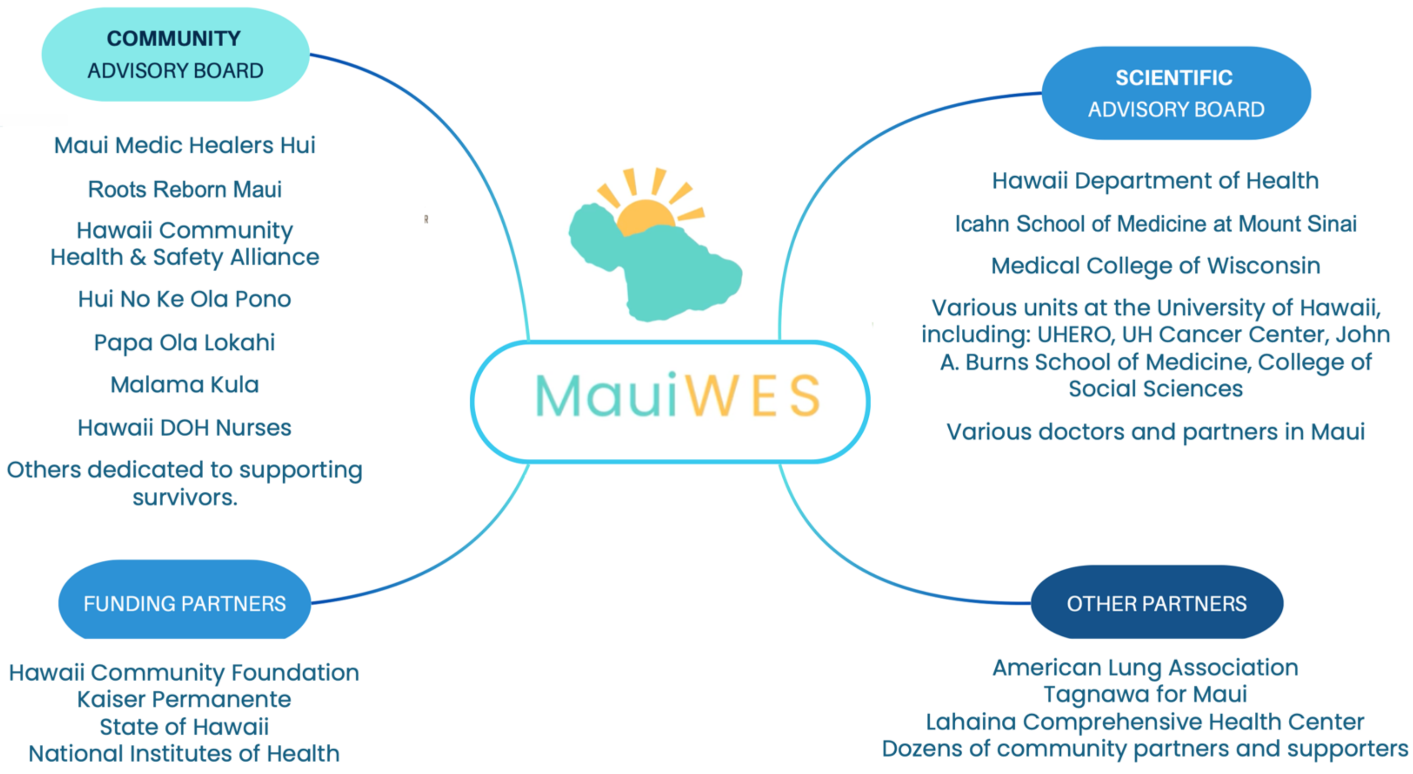

© 2025 Juarez R et al. *JAMA Network Open*.

1. Community Advisory Board. The Community Advisory Board (CAB) is a diverse group composed of Hispanic, NHPI, and Filipino representatives, formed in collaboration with our community partners. Members of the CAB have a deep understanding of the value of scientific research, biospecimen collection, and indigenous land and cultural considerations in Hawaii. This group has been actively involved in community-led recovery efforts in Maui since the onset of the wildfires, ensuring that the study aligns with the needs and concerns of affected populations.

The CAB will play a critical advisory role throughout the study, ensuring that recruitment, data collection, and dissemination strategies are culturally appropriate and respectful of community values. Specifically, the CAB will:

- Advise on community engagement strategies to optimize recruitment and retention.
- Ensure ethical handling of biospecimens, respecting Indigenous and cultural perspectives (see Data Management and Sharing).
- Review and guide data collection and privacy protocols to ensure trust and compliance with community expectations.
- Facilitate dissemination of research findings through culturally relevant channels, ensuring transparency and accessibility.
- Address emerging issues during the course of the study, providing community-driven solutions.

2. Scientific Advisory Board. The Scientific Advisory Board (SAB) consists of leading experts in environmental health, pediatric research, disaster epidemiology, and community-based participatory research. The SAB will provide technical and ethical oversight, ensuring that the study's design, implementation, and dissemination adhere to the highest scientific standards.

The primary roles of the SAB include:

- Reviewing study methodology to optimize data collection and analysis.
- Advising on biomarker and environmental exposure assessments to maximize scientific rigor.
- Ensuring ethical compliance and research integrity in the handling of participant data and biospecimens.
- Providing guidance on longitudinal data interpretation, particularly regarding pediatric health trajectories post-disaster.
- Supporting dissemination strategies by ensuring findings are communicated effectively to scientific, policy, and public health audiences.

3. Key Collaborating Partners. MauiWES integrates expertise from national and local organizations dedicated to supporting wildfire survivors. These partnerships ensure the study remains interdisciplinary, translational, and action-oriented. There are more than 50 collaborating partners, many who attended our community dissemination events, support us with space, staff, advocacy, etc. For brevite, we don't include all but some of them include: the American Lung Association, Hawaiʻi Integrated Analytics, Hawaiʻi Department of Health, or Tagnawa for Maui among others. These partners also provide additional research expertise, medical support, and outreach capacity, ensuring that MauiWES findings translate into real-world health improvements and policy interventions.

4. Funding Partners. MauiWES is supported by multiple funding agencies committed to advancing disaster research, community health resilience, and policy-driven recovery efforts.

Funding Agencies:

- State of Hawaiʻi
- National Institutes of Health (NIH/NIMHD)
- Hawaiʻi Community Foundation
- Kaiser Permanente

These funding partners enable large-scale data collection, community engagement initiatives, and direct health interventions, ensuring the study's long-term sustainability and impact.

© 2025 Juarez R et al. *JAMA Network Open*.

eMethods. Eligibility Criteria and Wildfire Exposure Score Computation and Methodology

Inclusion Criteria

Participants must meet all the following criteria to be eligible for enrollment in the Maui Wildfire Exposure Study (MauiWES):

1. **Age:** Must be an adult at the time of enrollment.
2. **Residency or Employment:** Must have lived or worked within 10 miles of Lahaina, Kula, or any other area directly affected by the August 2023 wildfires.
3. **Language Proficiency:** Must be able to speak and read at least one of the following languages: English, Tagalog, Ilocano, Hawaiian, Spanish, Samoan, Marshallese, Micronesian, Tongan, or Chamorro.
4. **Residency Duration & Commitment:** Must have resided in Maui for at least one year before the wildfires and be willing to participate in annual follow-ups for the next five years.
5. **In-Person Participation:** Must be able to attend a community-based event for health assessments, including biometric measurements, biospecimen collection, and a medical consultation.

Exclusion Criteria

Participants will be excluded if they meet any of the following conditions:

1. **Inability or unwillingness to participate**:
    - Unable or unwilling to complete an online questionnaire (or a paper questionnaire upon request).
    - Unable or unwilling to provide biospecimens.
2. **Medical Conditions**:
    - Diagnosed with a medical condition that would prevent full participation (e.g., uncontrolled major psychiatric disorders).
3. **Institutionalization**:
    - Currently residing in an institutional setting, such as a rehabilitation hospital or correctional facility.
4. **Relocation Plans**:
    - Planning to move away from Hawaii within the next 60 months (5 years).

© 2025 Juarez R et al. *JAMA Network Open*.

Wildfire Exposure Score Computation and Methodology

To quantify wildfire exposure and its impact on health outcomes, we developed four cumulative exposure scores that integrate residential location, exposure duration, and personal protective equipment (PPE) use across multiple time periods. Each score was computed and standardized on a 0–1 scale to facilitate meaningful comparisons and robust statistical modeling.

1. Fire Perimeter Classification and Exposure Severity Metrics

Wildfire exposure was first categorized based on residential location during the wildfire event, determined using satellite imagery, validated fire damage assessments, and geospatial modeling. Participants were classified into one of three groups:

- Inside the fire perimeter – Individuals who lived within the officially mapped burn zone.
- Outside the fire perimeter or not at home during the wildfire – Individuals residing in areas unaffected by direct fire damage or who were away from their home at the time of the fire.

To further quantify exposure duration, self-reported data on smoke, ash, and airborne debris exposure were collected across three key time periods:

1. Acute wildfire phase (August 8–11, 2023)
2. Immediate post-wildfire phase (August 12–September 30, 2023)
3. Post-wildfire exposure (October 2023–September 2024, tracked at three-month intervals)

2. Acute Exposure Score (August 8–11, 2023)

Participants reported the total duration of their exposure to wildfire smoke, ash, and debris to create an *Acute Exposure Score* for each participant.

- No exposure (0 hours) → 0
- Less than 1 hour → 0.2
- 1–4 hours → 0.4
- 5–8 hours → 0.6
- 9–12 hours → 0.8
- More than 12 hours → 1

3. Post-wildfire Exposure Score (October 2023–September 2024)

Participants reported their exposure frequency during five post-wildfire periods, covering a full year after the disaster:

1. August 12 – September 30, 2023
2. October 1 – December 31, 2023
3. January 1 – March 31, 2024

© 2025 Juarez R et al. *JAMA Network Open*.

4. April 1 – June 30, 2024
5. July 1 – September 30, 2024

For each time period, participants selected one of the following exposure frequency categories:

- "Never returned" → 0
- "Once per month" → 0.2
- "2–3 times per month" → 0.4
- "Once per week" → 0.6
- "2–3 times per week" → 0.8
- "Daily" → 1

Each participant's exposure for scores were normalized to the 0-1 range for each period and summed across the five post-fire periods to create the *Post-Wildfire Exposure Score* (0-5).

3. Personal Protective Equipment (PPE) Scores

To account for the potential mitigating effects of PPE use, participants reported how frequently they wore protective respiratory equipment (e.g., N95 masks, respirators) during both the wildfire period and post-wildfire exposure phases.

Wildfire Period PPE Score (August 8–11, 2023)

- "All/Most of the time" → 1
- "Some of the time" → 0.5
- "None of the time" → 0

Post-wildfire PPE Score (October 2023–September 2024)

For each of the five post-wildfire periods, PPE use was categorized as:

- "All/Most of the time" → 1
- "Some of the time" → 0.5
- "None of the time" → 0

A *Post-wildfire PPE Score* was calculated by summing PPE usage by normalizing each period's score to a 0–1 scale and summing them across all five post-fire periods (0–5).

Expanded Exposure Assessment Details

© 2025 Juarez R et al. *JAMA Network Open*.

eFigure 1. Maui Wildfire Perimeter and Participant Locations

Geospatial map of the wildfire perimeter, illustrating the locations of participants' households at the time of the fire in relation to the burn area.

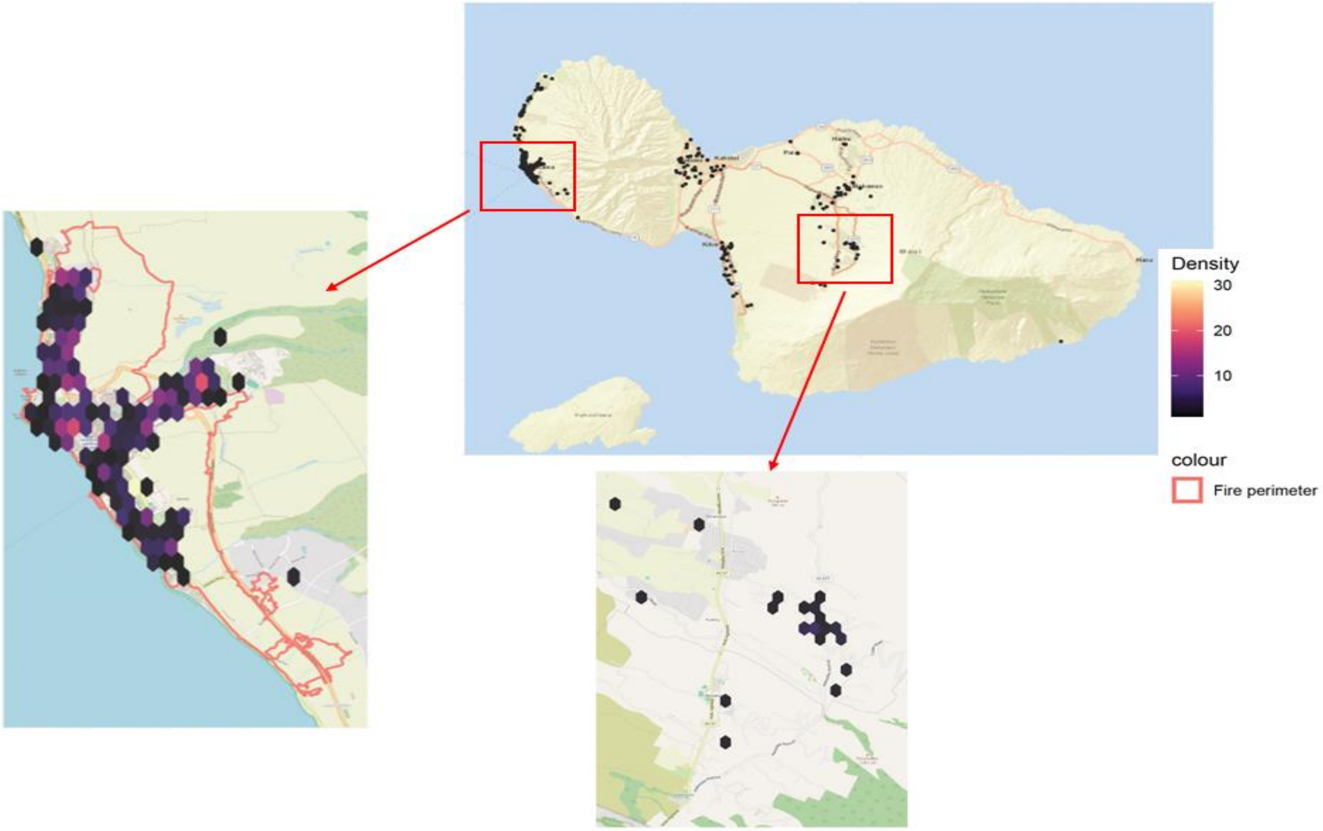

© 2025 Juarez R et al. *JAMA Network Open*.

5. Participant flow diagram

eFigure 2. Participant Flow Diagram for the MauiWES Dataset

The inclusion and exclusion of participants at each stage of the study. Of 1174 enrolled participants, 416 completed the follow-up questionnaire and were included in the MauiWES-ExpQ subsample. A total of 196 participants with A–C grade spirometry were included in the pulmonary analysis. Participants were excluded due to lack of follow-up (n = 758) or poor-quality spirometry (n = 220).

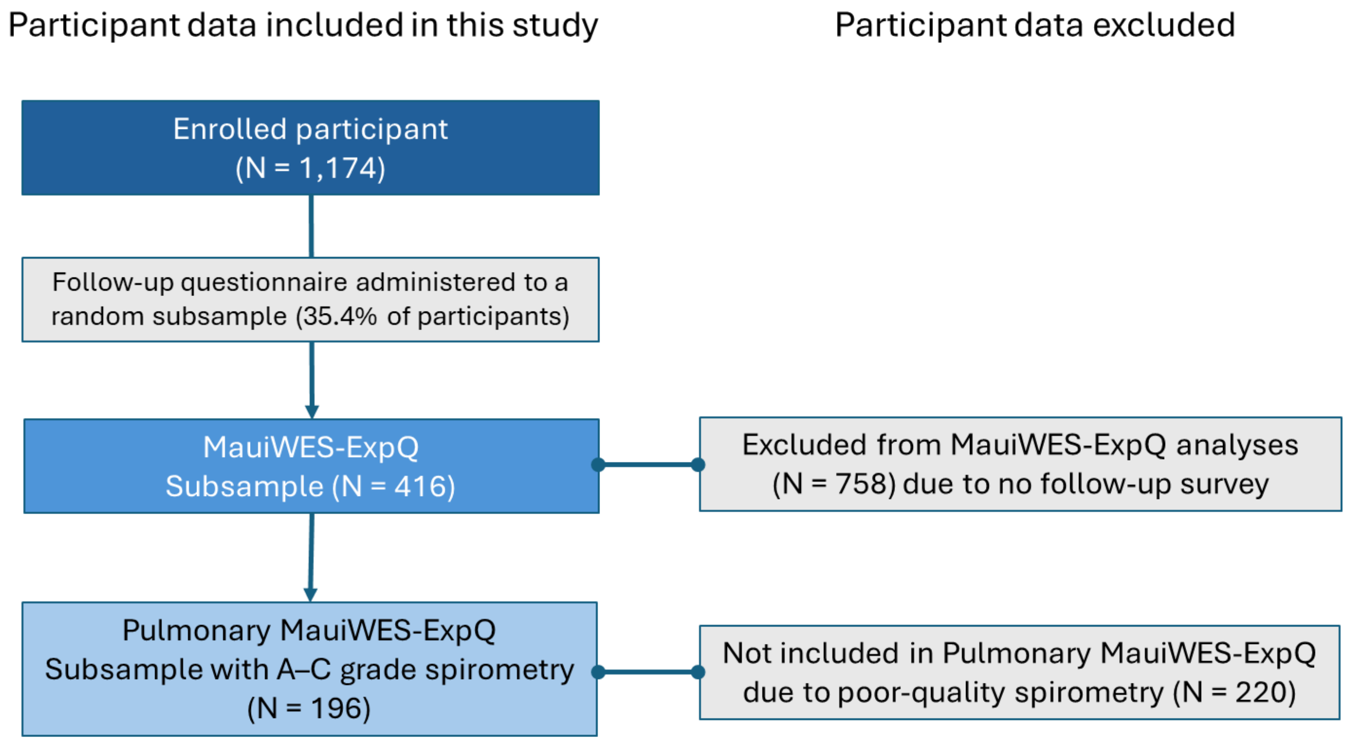

© 2025 Juarez R et al. *JAMA Network Open*.

eTable 1. Participant Characteristics of the MauiWES Cohort

Demographic, socioeconomic, and health characteristics of the MauiWES, MauiWES-ExpQ participants and Pulmonary MauiWES-ExpQ restricted to A, B, C spirometry quality.

	Full Cohort<br>N = 1,174	MauiWES-ExpQ<br>N = 416	p-value	Pulmonary MauiWES-ExpQ<br>N = 196	p-value
Age, Median (IQR)	47 (36 – 59)	44 (35 – 59)	0.42	43 (35 – 55)	0.19
Gender, n (%)			0.012		0.48
Male	462 (40)	132 (32)		53 (27)	
Female	684 (59)	279 (67)		140 (71)	
Other	11 (1.0)	5 (1.2)		3 (1.5)	
(Missing/no reported)	17	0			
Race, n (%)			0.01		>0.99
Asian	108 (9.3)	46 (11)		20 (10)	
Filipino	239 (20)	85 (20)		42 (22)	
Hispanic/Latino	203 (17)	51 (12)		22 (11)	
Native Hawaiian or Pacific Islander	238 (20)	66 (16)		33 (17)	
White	343 (29)	154 (37)		73 (37)	
Other	35 (3.0)	13 (3.1)		5 (2.6)	
(Missing/no reported)	8	1		1	
Income, n (%)			0.007		>0.99
At or Above FPL	580 (69)	234 (77)		112 (78)	
Below FPL	263 (31)	69 (23)		32 (22)	
(Missing/no reported)	331	113		52	
Food Insecurity, n (%)			0.57		0.67
Food secure	495 (47)	195 (50)		92 (50)	
Low food security	303 (29)	113 (29)		59 (32)	
Very low food security	245 (23)	82 (21)		34 (18)	
(Missing/no reported)	131	26		11	
Difficulty accessing healthcare, n (%)			0.27		0.82
Before and since fires	125 (11)	33 (8.4)		13 (7.0)	
No difficulty	736 (67)	272 (69)		128 (69)	
Only since fires	243 (22)	88 (22)		44 (24)	
(Missing/no reported)	70	23		11	
Number of participants displaced, n (%)	645 (56)	209 (50)	0.079	96 (49)	0.89
Employment status, n (%)			0.054		0.71
Employed	693 (59)	263 (63)		130 (67)	
Not employed - Looking for work	219 (19)	56 (13)		25 (13)	
Retired/Not employed-Not looking for work	258 (22)	96 (23)		40 (21)	
(Missing/no reported)	4	1		1	
Having health insurance, n (%)	998 (87)	376 (92)	0.011	179 (93)	0.78
BMI, Median (IQR)	28 (24 – 33)	27 (24 – 33)	0.63	28 (24 – 33)	0.54
(Missing/no reported)	24	8		1	
Self-rated health status, n (%)			0.25		0.97
Poor	62 (5.3)	21 (5.1)		9 (4.7)	
Fair	262 (23)	82 (20)		37 (19)	
Good	490 (42)	165 (40)		82 (42)	
Very good	273 (24)	120 (29)		53 (27)	
Excellent	72 (6.2)	23 (5.6)		12 (6.2)	
(Missing/no reported)	15	5		3	
Blood pressure, n (%)			0.92		0.93
Normal	315 (27)	113 (28)		57 (29)	
Elevated	112 (10)	36 (9)		17 (9)	
Stage 1 hypertension	490 (43)	178 (47)		83 (42)	
Stage 2 hypertension	235 (20)	81 (20)		39 (20)	
(Missing/no reported)	22	8		0	
FEV_1 (% predicted), Median (IQR)	95 (82 – 105)	96 (84 – 106)	0.17	95 (84 – 105)	0.65
(Missing/no reported)	504	170		0	
FVC (% perdicted), Median (IQR)	98 (87 – 108)	97 (87 – 107)	0.93	98 (88 – 107)	0.96
(Missing/no reported)	574	197		0	
FEV_1 session quality, n (%)			0.79		<0.001
A	474 (42)	169 (42)		143 (73)	
B	135 (12)	59 (15)		45 (23)	
C	63 (6)	18 (5)		8 (4)	
D	37 (3)	12 (3)		0 (0)	
E	318 (28)	104 (26)		0 (0)	
F	6 (1)	2 (1)		0 (0)	
U	91 (8)	34 (9)		0 (0)	
(Missing/no reported)	50	18		0	
FVC session qualtiy, n (%)			>0.99		<0.001
A	344 (31)	122 (31)		116 (59)	

© 2025 Juarez R et al. *JAMA Network Open*.

B	197 (18)	75 (19)		62 (32)	
C	62 (6)	22 (6)		18 (9)	
D	35 (3.1)	11 (2.8)		0 (0)	
E	297 (26)	104 (26)		0 (0)	
F	6 (0.5)	2 (0.5)		0 (0)	
U	183 (16)	62 (16)		0 (0)	
(Missing/no reported)	50	18		0	
Depression score, Median (IQR)	9 (5 – 15)	9 (5 – 15)	0.97	9 (5 – 15)	>0.99
(Missing/no reported)	64	12		8	
Depression, n (%)			0.47		0.98
Normal	556 (50)	210 (52)		97 (52)	
Depressive symptoms	445 (40)	149 (37)		69 (37)	
Highly depressive symptoms	109 (9.8)	45 (11)		22 (12)	
(Missing/no reported)	64	12		8	
Self-esteem score, Median (IQR)	20 (16 – 25)	21 (17 – 26)	0.18	20 (16 – 25)	0.60
(Missing/no reported)	63	15		8	
Self-esteem, n (%)			0.36		0.80
High Self-esteem	230 (21)	78 (19)		41 (22)	
Normal Self-esteem	637 (57)	221 (55)		101 (54)	
Low self-esteem	244 (22)	102 (25)		46 (24)	
(Missing/no reported)	63	15		8	
Suicide ideation, n (%)	52 (4)	17 (4)	0.84	10 (5)	0.72
(Missing/no reported)	37	8		4	
Social Support, n (%)			0.31		0.70
High social support	690 (60)	258 (63)		127 (66)	
Medium social support	121 (11)	47 (12)		22 (12)	
Low social support	331 (29)	102 (25)		42 (22)	
(Missing/no reported)	32	9		5	
Anxiety score, Median (IQR)	6.0 (1 – 10)	6.0 (1.0 – 11)	0.63	6.0 (2 – 10)	0.83
(Missing/no reported)	29	5		3	
Anxiety, n (%)			0.58		0.73
Minimal Anxiety	471 (41)	172 (42)		78 (40)	
Mild Anxiety	362 (32)	123 (30)		66 (34)	
Moderate Anxiety	174 (15)	57 (14)		23 (12)	
Severe Anxiety	138 (12)	59 (14)		26 (13)	
(Missing/no reported)	29	5		3	
Days affected by health issue , Median (IQR)	1 (0 – 7)	2 (0 – 7)	0.33	2 (0 – 7)	0.77
(Missing/no reported)	2	0			
Time between the fire and collection, Median (IQR)	201 (186 – 363)	327 (186 – 375)	<0.001	361 (197 – 375)	0.17

© 2025 Juarez R et al. *JAMA Network Open*.

eTable 2. Exposure Characteristics of the MauiWES-ExpQ

Summary of wildfire exposure characteristics among MauiWES-ExpQ participants, including residential proximity to the burn area, presence at home during the fire, and exposure index measures.

Characteristic	N = 416
Fire Perimeter Status, n (%)	
Outside	253 (63)
Inside	146 (37)
Unknown	17
Acute Exposure Score, Median (IQR)	0.40 (0.20 – 0.80)
Unknown	37
Post-wildfire Exposure Score, Median (IQR)	0.00 (0.00 – 1.00)
Unknown	14
Acute PPE Score, Median (IQR)	0.00 (0.00 – 0.50)
Unknown	115
Post-wildfire PPE Score, Median (IQR)	0.00 (0.00 – 1.00)
Unknown	14

© 2025 Juarez R et al. *JAMA Network Open*.

eTable 3. Association Between Wildfire Exposure and Reported Physical and Respiratory Symptoms

Multivariable regression analysis examining the relationship between wildfire exposure and the prevalence of physical and respiratory symptoms among study participants. Models adjust for key covariates, including pre-existing conditions, socioeconomic factors, and healthcare access.

Variable	Any physical symptoms (1)	Any physical symptoms (2)	Respiratory symptoms (1)	Respiratory symptoms (2)
Within fire perimeter	1.21 (0.53, 2.77)	0.92 (0.51, 1.67)	1.46 (0.56, 3.92)	1.18 (0.57, 2.46)
Exposure during the fire	8.15 (2.55, 28.06)	4.54 (1.79, 11.90)	8.91 (2.51, 34.73)	7.18 (2.28, 24.04)
Exposure after the fire	0.92 (0.61, 1.38)	0.84 (0.60, 1.15)	1.58 (1.02, 2.50)	1.51 (1.05, 2.19)
PPE during the fire	1.18 (0.45, 3.10)	0.98 (0.46, 2.06)	1.04 (0.34, 3.08)	1.01 (0.39, 2.59)
PPE after the fire	1.17 (0.72, 1.95)	1.15 (0.78, 1.72)	0.99 (0.58, 1.67)	0.97 (0.62, 1.51)
Pre-existing lung condition	2.03 (0.93, 4.49)		3.17 (1.38, 7.54)	3.17 (1.54, 6.70)
Pre-existing mental health condition	1.67 (0.73, 3.91)		1.37 (0.53, 3.53)	
Pre-existing other condition	1.37 (0.63, 3.00)	2.43 (1.38, 4.32)	1.29 (0.49, 3.50)	
BMI	1.01 (0.95, 1.08)		1.05 (0.98, 1.12)	
Age	1.02 (0.99, 1.05)		1.02 (0.99, 1.05)	1.03 (1.00, 1.05)
Filipino	0.55 (0.19, 1.57)	0.44 (0.19, 0.99)	0.24 (0.04, 0.98)	
Hispanic/Latino or Other	0.67 (0.21, 2.03)	0.70 (0.31, 1.60)	0.71 (0.19, 2.46)	
Native Hawaiian or Pacific Islander	0.51 (0.17, 1.48)	0.32 (0.14, 0.70)	0.65 (0.19, 2.06)	
Other Asian	0.60 (0.18, 2.03)	0.58 (0.22, 1.55)	0.88 (0.23, 3.19)	
Female	1.82 (0.84, 4.00)	1.92 (1.07, 3.49)	1.57 (0.62, 4.05)	
Days between participation and fire	1.00 (1.00, 1.01)		1.00 (0.99, 1.00)	
Health insurance	0.56 (0.15, 2.01)		1.01 (0.24, 4.78)	
Difficult access to care - Before and since fires	1.71 (0.54, 5.64)	2.02 (0.77, 5.52)	2.81 (0.75, 10.41)	2.92 (0.96, 8.78)
Difficult access to care - Only since fires	2.69 (1.08, 7.04)	2.03 (1.05, 4.02)	3.37 (1.32, 9.00)	3.74 (1.68, 8.53)
Low food security	1.17 (0.52, 2.66)		0.65 (0.25, 1.67)	
Income to poverty ratio	1.14 (0.90, 1.46)		0.80 (0.61, 1.04)	0.82 (0.65, 1.03)
Social support score	0.99 (0.97, 1.01)		0.99 (0.97, 1.01)	
Constant	0.05 (0.00, 1.66)	0.22 (0.08, 0.55)	0.02 (0.00, 0.99)	0.01 (0.00, 0.06)
Observations	191	261	191	208
LR test	0	0	0	0
Nagelkerke's Pseudo R2	0.331	0.254	0.397	0.317

© 2025 Juarez R et al. *JAMA Network Open*.

eTable 4. Association Between Wildfire Exposure and Lung Function Parameters

Multivariable regression analysis assessing the impact of wildfire exposure on lung function, including forced expiratory volume in one second (FEV_1), forced vital capacity (FVC), the FEV_1/FVC ratio, and forced expiratory flow at 25-75% of FVC (FEF_{25-75}%). Models adjust for key covariates such as age, pre-existing respiratory conditions, and others.

Variable	FEV1 % Predicted (Model 1)	FEV1 % Predicted (Model 2)	FVC % Predicted (Model 1)	FVC % Predicted (Model 2)	FEV1/FVC % Predicted (Model 1)	FEV1/FVC % Predicted (Model 2)	FEF25-75 % Predicted (Model 1)	FEF25-75 % Predicted (Model 2)
Within fire perimeter	-5.04 (-11.42, 1.35)	-5.97 (-10.81, -1.14)	-5.63 (-12.70, 1.45)	-6.58 (-11.73, -1.43)	-2.84 (-8.06, 2.39)	-0.49 (-3.91, 2.93)	-8.81 (-26.03, 8.42)	-5.85 (-16.99, 5.29)
Exposure during the fire	-0.35 (-9.42, 8.72)	0.36 (-7.08, 7.81)	-3.70 (-13.82, 6.43)	-3.62 (-11.94, 4.71)	1.00 (-6.65, 8.65)	-4.33 (-9.91, 1.26)	6.16 (-19.04, 31.35)	-2.49 (-20.24, 15.26)
Exposure after the fire	1.32 (-1.94, 4.58)	1.45 (-1.01, 3.91)	1.53 (-1.66, 4.71)	1.10 (-1.40, 3.60)	1.12 (-1.49, 3.72)	1.44 (-0.37, 3.25)	1.36 (-7.22, 9.94)	4.02 (-1.67, 9.71)
PPE during the fire	-4.64 (-12.21, 2.92)	-4.83 (-11.01, 1.36)	-6.68 (-14.80, 1.43)	-0.47 (-7.05, 6.10)	0.93 (-5.15, 7.01)	-1.59 (-6.12, 2.94)	-7.66 (-27.69, 12.37)	-9.32 (-23.36, 4.72)
PPE after the fire	-1.00 (-4.51, 2.50)	-0.78 (-3.72, 2.16)	-1.66 (-5.39, 2.07)	0.46 (-2.65, 3.57)	-0.36 (-3.15, 2.43)	-0.04 (-2.17, 2.09)	-4.15 (-13.36, 5.05)	-2.80 (-9.39, 3.78)
Pre-existing lung condition	-4.61 (-10.50, 1.28)	-7.46 (-12.32, -2.59)	-0.07 (-6.55, 6.42)		-3.73 (-8.41, 0.95)		-13.00 (-28.43, 2.42)	-14.51 (-25.55, -3.47)
Pre-existing mental health condition	-5.66 (-12.25, 0.93)		-2.87 (-9.99, 4.25)		0.63 (-5.04, 6.30)		-4.15 (-22.83, 14.54)	
Pre-existing other condition	-0.18 (-6.63, 6.28)		-0.31 (-7.90, 7.29)		0.05 (-5.69, 5.78)		-4.42 (-23.31, 14.47)	
BMI	0.43 (-0.03, 0.88)	0.35 (0.00, 0.70)	0.17 (-0.31, 0.65)		0.18 (-0.17, 0.53)		0.88 (-0.29, 2.04)	
Age	-0.23 (-0.45, -0.02)	-0.14 (-0.30, 0.02)	-0.07 (-0.30, 0.16)		-0.18 (-0.35, -0.01)		-0.39 (-0.95, 0.17)	
Filipino	-15.94 (-24.18, -7.71)	-12.74 (-19.38, -6.09)	-14.60 (-23.67, -5.54)	-8.42 (-15.22, -1.62)	0.56 (-6.07, 7.18)		-9.84 (-31.68, 11.99)	-11.39 (-25.80, 3.03)
Hispanic/Latino or Other	1.76 (-6.99, 10.51)	2.83 (-4.30, 9.95)	6.51 (-2.59, 15.61)	5.01 (-2.18, 12.21)	-3.55 (-10.42, 3.33)		-6.25 (-28.91, 16.41)	-0.09 (-16.04, 15.87)
Native Hawaiian or Pacific Islander	-7.82 (-16.34, 0.70)	-9.03 (-15.52, -2.55)	1.91 (-7.09, 10.91)	-2.95 (-9.61, 3.71)	-8.21 (-15.41, -1.01)		-26.61 (-50.34, -2.89)	-14.12 (-28.77, 0.52)
Other Asian	-11.38 (-19.99, -2.77)	-8.54 (-16.26, -0.83)	-8.05 (-17.72, 1.61)	-7.59 (-16.25, 1.08)	-2.67 (-9.78, 4.45)		-15.53 (-38.97, 7.91)	-17.15 (-35.43, 1.14)
Observations	117	164	100	153	92	138	92	138
LR test	0	0	0.003	0.004	0.032	0.171	0.054	0.015
Adjusted R²	0.214	0.24	0.177	0.094	0.102	0.02	0.079	0.081

© 2025 Juarez R et al. *JAMA Network Open*.

eTable 5. Association Between Wildfire Exposure and Days Impacted by Health Issues
Multivariable regression analysis examining the relationship between wildfire exposure and the number of days participants experienced health-related disruptions. Models adjust for key covariates, including pre-existing health conditions, socioeconomic factors, access to care, and social support.

Variable	Days Affected by Health Issues (1)	Days Affected by Health Issues (2)
Within fire perimeter	-1.07 (-3.99, 1.86)	-0.02 (-2.16, 2.12)
Exposure during the fire	16.52 (2.45, 30.58)	20.03 (8.87, 31.18)
Exposure after the fire	0.52 (-0.88, 1.91)	1.10 (0.01, 2.19)
PPE during the fire	1.86 (-1.54, 5.27)	0.37 (-2.33, 3.07)
PPE after the fire	0.81 (-0.86, 2.48)	0.41 (-0.93, 1.75)
Pre-existing lung condition	1.34 (-1.41, 4.08)	
Pre-existing mental health condition	2.38 (-0.55, 5.31)	2.73 (0.41, 5.05)
Pre-existing other condition	3.43 (0.51, 6.36)	3.25 (1.12, 5.39)
BMI	0.14 (-0.07, 0.35)	
Age	0.00 (-0.10, 0.11)	
Filipino	-2.62 (-6.47, 1.23)	
Hispanic/Latino or Other	-1.20 (-5.12, 2.73)	
Native Hawaiian or Pacific Islander	-1.65 (-5.52, 2.22)	
Other Asian	-2.72 (-7.11, 1.66)	
Female	-0.96 (-3.73, 1.82)	
Days between participation and fire	-0.00 (-0.02, 0.01)	
Health insurance	-1.55 (-6.03, 2.93)	
Difficult access to care - Before and since fires	-1.24 (-5.37, 2.90)	
Difficult access to care - Only since fires	1.19 (-1.99, 4.37)	
Low food security	3.81 (0.90, 6.73)	3.16 (1.07, 5.26)
Income to poverty ratio	0.39 (-0.42, 1.20)	
Social support score	0.01 (-0.14, 0.16)	0.05 (-0.07, 0.16)
Social support score * Exposure during the fire	-0.19 (-0.41, 0.03)	-0.25 (-0.43, -0.08)
Constant	-3.68 (-18.39, 11.04)	-4.27 (-12.33, 3.78)
Observations	191	259
LR test	0	0
Adjusted R2	0.212	0.208

© 2025 Juarez R et al. *JAMA Network Open*.

eTable 6. Association Between Wildfire Exposure and Mental Health Outcomes
Multivariable regression analysis examining the relationship between wildfire exposure and mental health outcomes, including depression (PHQ-9) and anxiety (GAD-7) scores. Models adjust for key covariates such as pre-existing health conditions, socioeconomic factors, social support, and access to care

Variable	Depression Score (1)	Depression Score (2)	Anxiety Score (1)	Anxiety Score (2)
Within fire perimeter	-1.19 (-4.19, 1.80)	0.47 (-1.29, 2.22)	-1.09 (-3.70, 1.52)	0.99 (-0.55, 2.54)
Exposure during the fire	7.60 (-3.67, 18.87)	9.43 (0.61, 18.25)	6.50 (-3.32, 16.32)	1.57 (-0.88, 4.01)
Exposure after the fire	0.34 (-0.79, 1.47)	0.29 (-0.60, 1.17)	0.96 (-0.02, 1.94)	0.84 (0.02, 1.65)
PPE during the fire	1.41 (-1.43, 4.24)	0.30 (-1.88, 2.47)	0.78 (-1.69, 3.24)	-0.79 (-2.74, 1.16)
PPE after the fire	0.26 (-1.11, 1.63)	0.28 (-0.80, 1.35)	-0.11 (-1.30, 1.08)	-0.21 (-1.19, 0.77)
Pre-existing lung condition	1.37 (-0.85, 3.59)		0.71 (-1.21, 2.64)	
Pre-existing mental health condition	2.14 (-0.24, 4.51)	2.19 (0.29, 4.09)	1.99 (-0.07, 4.05)	1.80 (0.12, 3.49)
Pre-existing other condition	2.33 (-0.03, 4.70)	2.17 (0.44, 3.91)	2.06 (0.01, 4.11)	2.47 (0.93, 4.02)
BMI	-0.02 (-0.19, 0.15)		-0.01 (-0.16, 0.14)	
Age	0.01 (-0.08, 0.10)		-0.04 (-0.11, 0.04)	
Filipino	-1.24 (-4.37, 1.88)	-1.35 (-3.87, 1.16)	-0.48 (-3.20, 2.25)	
Hispanic/Latino or Other	-1.38 (-4.54, 1.79)	-1.21 (-3.66, 1.25)	0.61 (-2.15, 3.36)	
Native Hawaiian or Pacific Islander	-2.45 (-5.65, 0.76)	-3.72 (-6.10, -1.35)	-0.21 (-3.00, 2.59)	
Other Asian	-2.13 (-5.66, 1.40)	-1.80 (-4.82, 1.23)	-0.67 (-3.74, 2.40)	
Female	1.28 (-0.95, 3.51)	1.74 (0.02, 3.46)	1.21 (-0.73, 3.15)	
Days between participation and fire	-0.00 (-0.02, 0.01)		-0.01 (-0.02, 0.00)	
Health insurance	-0.57 (-4.19, 3.04)		1.12 (-2.03, 4.26)	
Difficult access to care - Before and since fires	0.13 (-3.18, 3.45)	0.33 (-2.54, 3.20)	1.33 (-1.57, 4.22)	1.85 (-0.77, 4.47)
Difficult access to care - Only since fires	2.24 (-0.33, 4.80)	2.52 (0.54, 4.50)	2.16 (-0.07, 4.38)	2.81 (1.08, 4.55)
Low food security	2.82 (0.41, 5.23)	3.39 (1.62, 5.15)	2.02 (-0.08, 4.12)	2.95 (1.46, 4.43)
Income to poverty ratio	0.29 (-0.37, 0.96)		-0.05 (-0.62, 0.53)	
Displaced	1.89 (-1.09, 4.88)		1.24 (-1.36, 3.85)	
Unemployed	0.94 (-2.24, 4.11)		1.50 (-1.20, 4.19)	
Retired or not in labor force	-0.93 (-3.87, 2.01)		-0.61 (-3.18, 1.96)	
Social support score	-0.04 (-0.15, 0.08)	-0.01 (-0.11, 0.08)	-0.02 (-0.13, 0.08)	
Social support score * Exposure during the fire	-0.08 (-0.26, 0.09)	-0.12 (-0.26, 0.01)	-0.06 (-0.22, 0.09)	
Constant	7.02 (-5.05, 19.09)	6.18 (-0.23, 12.58)	5.69 (-4.83, 16.20)	1.47 (-0.79, 3.74)
Observations	190	244	191	251
LR test	0	0	0	0
Adjusted R2	0.196	0.257	0.158	0.158

© 2025 Juarez R et al. *JAMA Network Open*.

eTable 7. Association Between Wildfire Exposure and Hypertension Risk
Multivariable regression analysis assessing the relationship between wildfire exposure and hypertension prevalence. Models adjust for key covariates, including pre-existing health conditions, BMI, age, socioeconomic factors, healthcare access, and social support. Findings explore potential mechanisms linking wildfire-related stressors and environmental exposures to cardiovascular health outcomes.

	Hypertension	Hypertension
Within fire perimeter	1.27 (0.56, 2.88)	1.12 (0.65, 1.94)
Exposure during the fire	0.99 (0.32, 3.13)	0.71 (0.29, 1.73)
Exposure after the fire	0.97 (0.66, 1.47)	0.99 (0.74, 1.36)
PPE during the fire	1.94 (0.75, 5.29)	2.22 (1.07, 4.78)
PPE after the fire	0.97 (0.60, 1.56)	0.98 (0.68, 1.43)
Pre-existing lung condition	0.80 (0.37, 1.73)	
Pre-existing mental health condition	0.91 (0.40, 2.07)	
Pre-existing other condition	1.17 (0.52, 2.68)	
BMI	1.15 (1.08, 1.23)	1.11 (1.06, 1.17)
Age	1.01 (0.98, 1.04)	1.02 (1.00, 1.04)
Filipino	2.18 (0.73, 6.98)	
Hispanic/Latino or Other	1.35 (0.45, 4.12)	
Native Hawaiian or Pacific Islander	0.73 (0.26, 2.08)	
Other Asian	1.91 (0.56, 7.20)	
Female	0.45 (0.20, 1.01)	
Days between participation and fire	1.00 (1.00, 1.00)	
Health insurance	2.96 (0.89, 10.34)	
Difficult access to care - Before and since fires	1.56 (0.49, 5.38)	1.56 (0.61, 4.38)
Difficult access to care - Only since fires	0.86 (0.36, 2.04)	0.95 (0.50, 1.80)
Low food security	0.82 (0.36, 1.82)	
Income to poverty ratio	0.97 (0.78, 1.21)	
Social support score	1.00 (0.98, 1.02)	
Constant	0.02 (0.00, 0.67)	0.03 (0.01, 0.19)
Observations	191	272
LR test	0.01	0.00
Nagelkerke's Pseudo R2	0.252	0.145

© 2025 Juarez R et al. *JAMA Network Open*.

eFigure 3. Regression Analysis of Pre-existing Conditions and Health Outcomes

Multivariable regression analysis examining the association between pre-existing health conditions and key post-wildfire health outcomes, including lung function (FEV_1, FVC), days affected by health issues, depression, and anxiety.

Pre-existing conditions

Table of regression coefficients

Variable label	Coef (95% CI)
Pre-existing lung condition	-7.46 (-12.32, -2.59)
Pre-existing mental health condition	2.73 (0.41, 5.05)
Other pre-existing condition	3.25 (1.12, 5.39)
Pre-existing mental health condition	2.19 (0.29, 4.09)
Other pre-existing condition	2.17 (0.44, 3.91)
Pre-existing mental health condition	1.80 (0.12, 3.49)
Other pre-existing condition	2.47 (0.93, 4.02)
Other pre-existing condition	2.43 (1.38, 4.32)
Pre-existing lung condition	3.17 (1.54, 6.70)

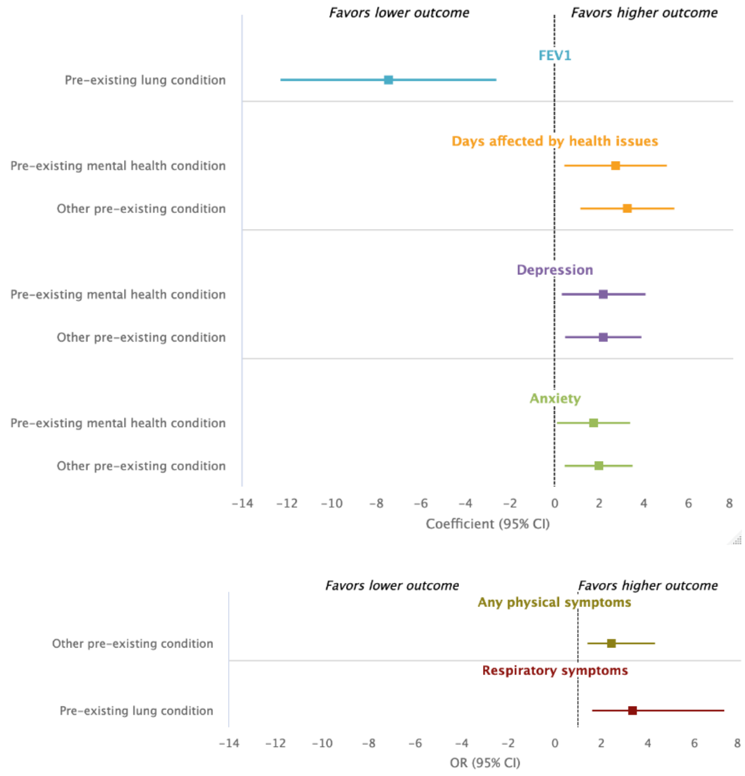

© 2025 Juarez R et al. *JAMA Network Open*.

eResults. Race and Ethnic Disparities in Health Outcomes

The impact of the wildfire on lung health, mental health, and overall well-being varied significantly across racial and ethnic groups. Regression analyses revealed disparities in lung function, with Filipino participants exhibiting the most pronounced reductions in FEV_1 (-12.74; 95% CI: -19.38, -6.09) and FVC (-8.42; 95% CI: -15.22, -1.62). Native Hawaiian or Pacific Islander participants also showed significant declines in FEV_1 (-9.03; 95% CI: -15.52, -2.55), whereas reductions among Other Asian groups were slightly lower (-8.54; 95% CI: -16.26, -0.83). In contrast, Hispanic/Latino or Other participants did not exhibit statistically significant reductions in FEV_1 (2.83; 95% CI: -4.30, 9.95) or FVC (5.01; 95% CI: -2.18, 12.21), suggesting potential differences in susceptibility or exposure levels.

Regarding mental health outcomes, Native Hawaiian or Pacific Islander participants reported significantly lower depression scores compared to other groups (-3.72; 95% CI: -6.10, -1.35), whereas Filipino (-1.35; 95% CI: -3.87, 1.16) and Other Asian (-1.80; 95% CI: -4.82, 1.23) participants did not exhibit statistically significant changes. These results suggest that cultural, social, and community support structures may have played a role in buffering mental health impacts for some groups more than others.

These findings highlight the importance of considering racial and ethnic disparities in wildfire-related health outcomes. Future interventions should be tailored to address the specific vulnerabilities of different communities, ensuring equitable access to healthcare, mental health services, and long-term recovery support.

© 2025 Juarez R et al. *JAMA Network Open*.

eFigure 4. Racial and Ethnic Disparities in Postwildfire Health Outcomes

Multivariable regression analysis examining differences in lung function (FEV_1, FVC) and mental health (depression scores) across racial and ethnic groups following wildfire exposure. Models adjust for key demographic and socioeconomic factors.

Race/ethnicity
Table of regression coefficients

Variable Label	Coef (95% CI)
Native Hawaiian or Pacific Islander	-9.03 (-15.52, -2.55)
Filipino	-12.74 (-19.38, -6.09)
Other Asian	-8.54 (-16.26, -0.83)
Hispanic/Latino or Other	2.83 (-4.30, 9.95)
Native Hawaiian or Pacific Islander	-2.95 (-9.61, 3.71)
Filipino	-8.42 (-15.22, -1.62)
Other Asian	-7.59 (-16.25, 1.08)
Hispanic/Latino or Other	5.01 (-2.18, 12.21)
Native Hawaiian or Pacific Islander	-3.72 (-6.10, -1.35)
Filipino	-1.35 (-3.87, 1.16)
Other Asian	-1.80 (-4.82, 1.23)
Hispanic/Latino or Other	-1.21 (-3.66, 1.25)

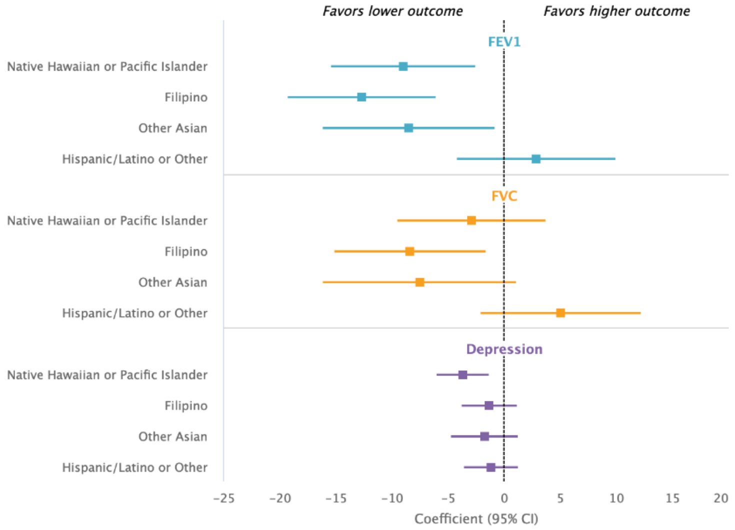

© 2025 Juarez R et al. *JAMA Network Open*.

eReferences

1. Federal Emergency Management Agency. Preliminary After-Action Report: 2023 Maui Wildfire. February 8, 2024. Accessed October 10, 2024. https://www.usfa.fema.gov/blog/preliminary-after-action-report-2023-maui-wildfire/

2. Sacks B. Months after Maui fires, residents report troubling health problems. The Washington Post. May 15, 2024. Accessed February 11, 2025. https://www.washingtonpost.com/climate-environment/2024/05/15/maui-fire-health-impact-lahaina/

3. U.S. Department of Health and Human Services Office of Minority Health. Diabetes and Native Hawaiians/Pacific Islanders. March 1, 2021. Accessed February 11, 2025. https://minorityhealth.hhs.gov/diabetes-and-native-hawaiianspacific-islanders#:~:text=In%202018%2C%20Native%20Hawaiians/Pacific,than%20the%20national%20white%20population.

4. Centers for Disease Control and Prevention. Disparities in Diabetes Prevalence Among Native Hawaiians/Other Pacific Islanders and Asians in Hawai‘i. February 21, 2019. Accessed October 20, 2024. https://www.cdc.gov/pcd/issues/2019/18_0187.htm

5. Stearnbourne C. The health impacts of wildfires: Frequently asked questions. Harvard T.H.Chan. School of Public Health. January 31, 2025. Accessed February 11, 2025. https://hsph.harvard.edu/environmental-health/news/health-impacts-of-wildfires-frequently-asked-questions/

6. US Environmental Protection Agency, EPA. Why Wildfire Smoke is a Health Concern. January 30, 2025. Accessed February 11, 2025. https://www.epa.gov/wildfire-smoke-course/why-wildfire-smoke-health-concern#:~:text=Fine%20particles%20(also%20known%20as,are%20of%20greatest%20health%20concern.

7. MauiWES. Maui Wildfire Exposure Study. 2023. Accessed February 1, 2025. https://www.mauiwes.info/

8. Public Health Institute. Maui Wildfire Exposure Study. Presented at: Public Health Institute Presentation; August 15, 2024. Accessed February 1, 2025. https://www.phi.org/wp-content/uploads/2024/09/MauiWES_Talk_August2024-compressed.pdf

9. University of Hawai‘i Economic Research Organization. *Maui Wildfire Exposure Study: Community Health, Wellbeing, and Resilience.* University of Hawai‘i Economic Research Organization.; 2024. Accessed February 1, 2025. https://uhero.hawaii.edu/wp-content/uploads/2024/05/MauiExposureStudy.pdf

10. MauiWES. The Maui Wildfire Exposure Study: View Survey Results. 2025. Accessed February 1, 2025. https://analytics.uhero.hawaii.edu/maui-wes

© 2025 Juarez R et al. *JAMA Network Open*.
