# Supplementary material for: Health and Social Support in the Aftermath of the Maui Wildfires
Source: JAMA Netw Open. 2025 Aug 6;8(8):e2525430. doi: 10.1001/jamanetworkopen.2025.25430 (PMC12329611; doi:10.1001/jamanetworkopen.2025.25430)
Supplement: Supplement 2. — Data Sharing Statement [file jamanetwopen-e2525430-s002.pdf]

Data Sharing Statement

Juarez. Health and Social Support in the Aftermath of the Maui Wildfires. *JAMA Netw Open*. Published August 06, 2025. doi:10.1001/jamanetworkopen.2025.25430

Data
Data available: Yes
Data types: Deidentified participant data
How to access data: At our study website MauiWES.org
When available: With publication

Supporting Documents
Document types: None

Additional Information
Who can access the data: researchers whose proposed use of the data has been approved
Types of analyses: for any purpose benefiting the community
Mechanisms of data availability: with a signed data access agreement
